# 1D TiO_2_ photoanodes: a game-changer for high-efficiency dye-sensitized solar cells

**DOI:** 10.1039/d4ra06254j

**Published:** 2025-02-13

**Authors:** Kumar Vaisno Srivastava, Pooja Srivastava, Akancha Srivastava, Raj Kumar Maurya, Yatendra Pal Singh, Abhishek Srivastava

**Affiliations:** a Department of Physics, Mangalayatan University Aligarh 202146 India; b Department of Physics, Dr RML Avadh University Ayodhya 224001 India

## Abstract

Hierarchical architectures encompassing one-dimensional (1D), two-dimensional (2D), and three-dimensional (3D) nanostructures have garnered significant attention in energy and environmental applications due to their unique structural, electronic, and optical properties. These architectures provide high surface area, enhanced charge transport pathways, and improved light-harvesting capabilities, making them versatile candidates for next-generation photovoltaic (PV) systems. Among these, 1D structures, such as nanorods, nanowires, and nanotubes, offer distinct advantages, including anisotropic charge transport, reduced recombination rates, and enhanced light absorption due to their high aspect ratio and directional charge flow. In this focused review article, the pivotal role of one-dimensional titanium dioxide (1-D TiO_2_) as photoanodes in dye-sensitized solar cells (DSSCs) has been discussed thoroughly. The distinctive morphology of 1-D TiO_2_, including nanotubes or nanorods, provides an expanded surface area, facilitating efficient light absorption and dye adsorption. The inherent one-dimensional architecture promotes accelerated electron transport, minimizing recombination losses and enhancing charge collection efficiency. Additionally, 1-D TiO_2_ structures exhibit superior charge carrier mobility, reducing trapping sites and enhancing electron diffusion pathways, thereby improving overall stability and performance. The scalability and cost-effectiveness of synthesizing 1-D TiO_2_ nanostructures underscore their potential for large-scale DSSC production. This research emphasizes the significance of 1-D TiO_2_ as a promising photoanode material, offering a pathway for advancing the efficiency and viability of dye-sensitized solar cell applications.

## Abstract 2

A schematic illustrating the initial characteristics, phases, nanostructures, and potential future applications of 1D TiO_2_ photoanodes.

## Graphical abstract

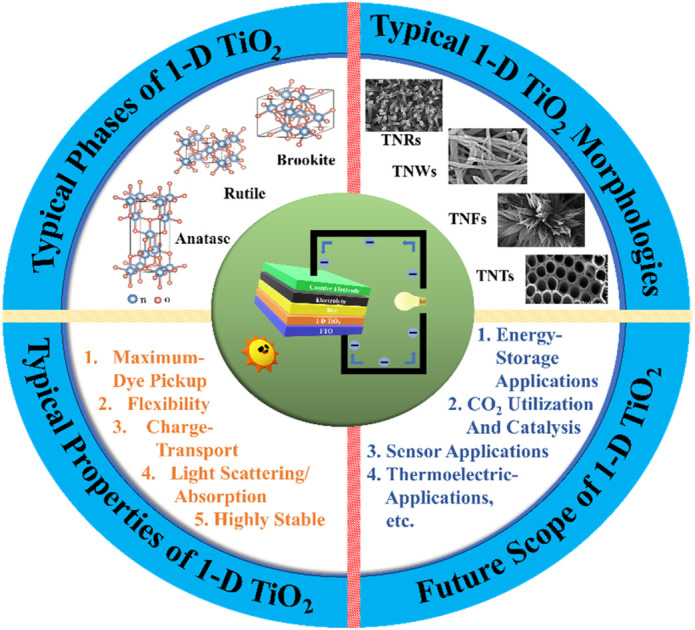

## 1. Introduction

The excessive use of fossil fuels to meet our growing energy needs has resulted in significant consequences such as global warming and environmental pollution. To address these challenges and achieve a sustainable energy future, it is crucial to adopt an energy production approach that relies on renewable sources. While options like geothermal energy, biofuels, and wind-tidal energies can contribute to sustainable development, solar energy stands out as a particularly important solution. In this era of increasing energy demands and environmental concerns, solar radiation offers immense potential, abundant availability, and environmental friendliness.^1^ Out of the 4 million exajoules (1018 J) of solar energy reaching the Earth, approximately 50 000 EJ can be easily harnessed. The significance of solar energy is underscored by the fact that the world's annual energy consumption in 2017 amounted to approximately 565 exajoules (EJ), which represents a mere fraction of the potential solar energy harvest of around 50 000 EJ. However, the current contribution of the renewable energy sector to global electricity production stands at a modest 8.4%. Notably, solar energy accounts for over 20% of this renewable energy share.^2^ Despite this, the utilization of solar energy remains disproportionately small compared to the vast solar power available for exploitation. The primary reason for this disparity lies in the absence of efficient and economically viable solar harvesting techniques. Among the diverse solar energy conversion devices, first-generation silicon solar cells have gained prominence as the most extensively commercialized type.

Silicon solar cells have achieved efficiencies of approximately 26%, approaching the theoretical maximum efficiency. In contrast, second-generation solar cells utilize thin-film technology and employ materials like CdTe and CIGS, reaching efficiencies of up to 21.7% in recent developments.^3–5^ However, the widespread commercialization of first and second-generation solar cells remains costly, and the materials used in these generations can pose environmental hazards. To address these challenges and create an economically viable and environmentally friendly approach to solar energy conversion, the concept of dye-sensitized solar cells (DSSCs) was introduced as the third generation of solar cells. DSSCs, first proposed by Michael Grätzel in 1991, have garnered significant attention due to their potential for large-scale, cost-effective production and flexibility.^6^ Currently, the efficiencies of DSSCs are within the range of 14.3%.^7^ DSSCs have the potential to play a crucial role in advancing sustainable energy culture if the challenges of low efficiency and limited durability are effectively addressed.

The large-scale commercialization of DSSCs can harness a significant portion of the available solar radiation. Enhancing performance, improving durability, and reducing production costs of DSSCs can be achieved through various approaches. This involves synthesizing novel component materials or modifying existing materials and introducing more optimized DSSC designs.^8^ As DSSC assemblies consist of multiple components such as the photoanode, sensitizer (dye), counter electrode, and redox electrolyte, efforts to enhance performance can be focused on any of these components.^9^ This review specifically highlights the significance of 1-D TiO_2_ nanorods-based photoanodes for the critical advancement of DSSCs. Specifically, 1D nanorods were selected due to their superior directional charge transport, enhanced light scattering properties, and higher surface area-to-volume ratio, which collectively improve photocatalytic and photoelectrochemical performance. Despite their high surface area, nanoparticles suffer from significant interparticle recombination and poor charge transport due to grain boundaries, limiting their overall efficiency.^10^ Nanowires and nanofibers, while offering continuous 1-D pathways for charge transport, have lower surface areas, which can restrict dye loading and limit their light-harvesting capability. In contrast, nanorods strike an optimal balance between surface area and charge transport. Their unidirectional structure facilitates efficient electron transport with reduced recombination compared to nanoparticles, while their higher surface area relative to nanowires enables improved dye loading and light absorption.^11^ Additionally, the tunable aspect ratio of nanorods allows for enhanced light scattering and extended photon absorption paths, making them highly effective as photoanodes. This provides a robust background for the study and highlights the rationale behind using nanorods as the preferred architecture for DSSC applications.^12^

Additionally, heterojunction solar cells, compared to DSSCs, have many demerits. Thin-film solar cells suffer from higher charge recombination rates due to the presence of multiple interfaces, which act as recombination centers, thereby reducing charge carrier lifetime and overall efficiency. Additionally, their thin active layers, typically less than a few hundred nanometres, limit light absorption, particularly in the red and near-infrared regions of the solar spectrum, resulting in suboptimal photon utilization.^13,14^ These devices also exhibit thermal and mechanical instability, with a higher tendency for delamination, cracking, and degradation under prolonged thermal or mechanical stress, which negatively impacts their long-term operational stability. Moreover, the fabrication of thin-film solar cells is complex, often requiring vacuum deposition, high-temperature processing, and precise control over layer thickness and composition, leading to higher production costs and limited scalability.^15,16^ In contrast, DSSCs offer several advantages, including simpler, solution-based fabrication methods, lower sensitivity to defects, and better performance under low-light and diffuse lighting conditions.

Therefore, this review explores the advancements and potential of 1-D TiO_2_ nanorods as a most promising photoanodes in DSSCs. Their superior light-scattering properties, efficient electron transport, and well-matched band alignment with sensitizers make them promising candidates for high-performance DSSCs. Additionally, 1-D TiO_2_ is favoured for its non-toxic nature and robust functionality in sustainable energy applications. Future research could focus on extending their light absorption into the infrared region and integrating them with advanced materials like perovskites, quantum dots, and MXenes to enhance energy capture, transfer, and storage. Addressing challenges related to scalability, surface engineering, and hybridisation could further position 1-D TiO_2_-based DSSCs as pivotal in driving the global transition to clean and sustainable energy solutions.

## 2. Fundamentals of DSSC

### 2.1 Architecture

**Fig. 1:**
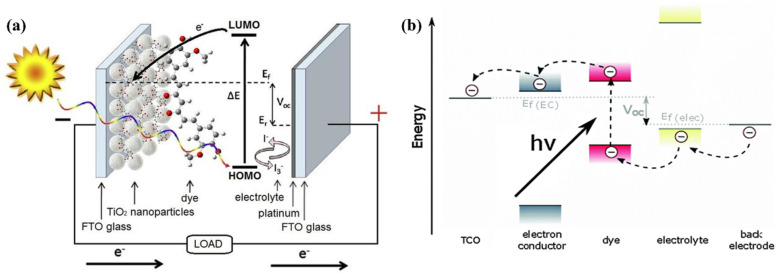
(a) Schematic assembly of DSSC and typical working mechanism,^17^ (b) typical band alignment of DSSC.

DSSCs are composed of several essential components that work together to convert sunlight into electricity, as shown in Fig. 1(a). The architecture begins with a transparent conductive substrate, typically made of fluorine-doped tin oxide (FTO) or indium tin oxide (ITO) on glass, which serves as the bottom electrode and allows light transmission. The photoanode, a thin layer of nanostructured TiO_2_ particles coated on the substrate, provides a large surface area for dye adsorption and facilitates electron transport. A light-absorbing sensitizer, such as ruthenium-based or organic dyes, is adsorbed onto the TiO_2_ surface to capture photons and inject excited electrons into the TiO_2_, initiating charge separation. A liquid electrolyte containing a redox couple (usually iodide/triiodide) facilitates charge transport by shuttling electrons between the photoanode and the counter electrode. The counter electrode, commonly made of platinum or carbon-based materials, catalyzes the reduction of the oxidized redox species and acts as the cathode. A transparent conductive layer on the counter electrode, often FTO or ITO, ensures efficient electron collection to complete the circuit. A sealing material such as glass or polymer is used for encapsulation to protect the cell from environmental factors.^18,19^ When sunlight strikes the cell, the dye absorbs photons, resulting in electron excitation and injection into the TiO_2_. These electrons flow through the external circuit to the counter electrode, reducing the redox couple in the electrolyte and completing the electrical cycle. The unique architecture of DSSCs enables efficient light absorption, charge separation, and electron collection, making them a promising technology for solar energy conversion. Ongoing research aims to refine the architecture, explore novel materials, and improve both the efficiency and stability of these cells.^20,21^

### 2.2 Working principal

The working principle of DSSCs involves several key processes enabling sunlight-to-electricity conversion, as shown in Fig. 1(b). Upon illumination, the photoanode, composed of nanostructured TiO_2_ particles coated with dye molecules (sensitizers), absorbs light. Photons excite electrons in the dye to higher energy states, which are subsequently injected into the conduction band of TiO_2_ due to favourable energy level alignment (eqn (1) and (2)). This creates charge separation, with electrons traveling through the TiO_2_ network while oxidized dye molecules are regenerated *via* redox reactions in the electrolyte (eqn (3)). The electrolyte, containing an iodide/triiodide (I^−^/I_3_^−^) redox couple, facilitates charge balance by reducing I_3_^−^ to I^−^at the photoanode and regenerating I_3_^−^ at the counter electrode.

1 S + *hν* → S*

2 S^*^ → S^+^ + e^−^ (TiO_2_)

3 2S^+^ + 3I^−^ → 2S + I_3_^−^

4 I_3_^−^ + 2e^−^ → 3I^−^

Electrons injected into TiO_2_ travel through a conductive collecting layer to the counter electrode, typically platinum (Pt), where they catalyze the reduction of I_3_^−^ to I^−^, completing the circuit and generating an electric current (eqn (4)). The continuous absorption of sunlight, charge separation, and electron transport ensure sustained electricity generation. DSSC efficiency is influenced by factors such as sensitizer properties, photoanode and counter-electrode design, and the effectiveness of light absorption and charge transport. Research is ongoing to optimize these components for improved performance and stability.^22–26^

### 2.3 Electrical modelling and key parameters

Electrical modeling of DSSCs involves representing their behavior using an equivalent circuit that includes components mimicking the physical and chemical processes occurring within the cell. The key elements of the model include a photocurrent source (*I*_ph_), a diode (*D*), a series resistance (*R*_S_), and a shunt resistance (*R*_Sh_). The photocurrent source, *I*_ph_, represents the current generated by the photoanode due to light absorption and subsequent electron injection into the TiO_2_ conduction band. It depends on factors like light intensity, dye absorption efficiency, and charge collection efficiency.

The diode models the recombination of photo-generated electrons with oxidized species in the electrolyte or the dye, with its current (*I*_D_) described by the Shockley equation:5
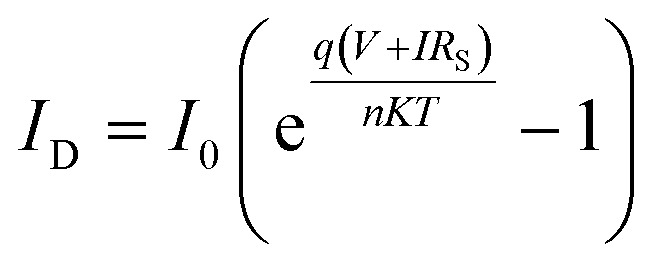
where *I*_0_ is the reverse saturation current, *q* is the elementary charge, *n* is the ideality factor, *k* is Boltzmann's constant, *T* is the temperature, *V* is the voltage across the cell, and *R*_S_ is the series resistance.

The *R*_S_ accounts for resistive losses in the TCO layer, the TiO_2_ film, and the electrolyte. A lower *R*_S_ improves the fill factor and overall efficiency of the cell. The *R*_Sh_ represents leakage currents due to defects or imperfections in the device. A high *R*_Sh_ is desirable to minimize losses and improve device performance. The relationship between the total current (*I*) and voltage (*V*) in the DSSC is given by:6
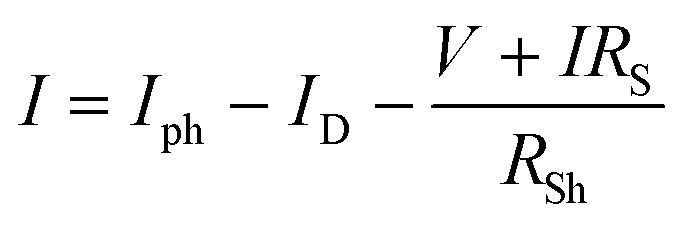


The power output of the cell can be derived as:7
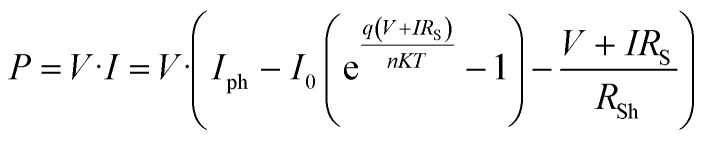


**Fig. 2:**
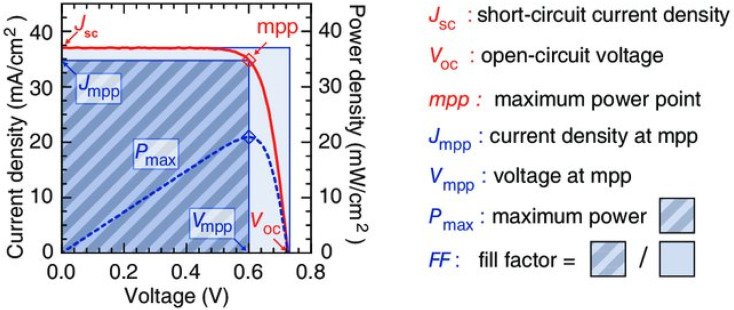
Typical schematic representation of various *J*–*V* parameters to evaluate the solar cell performance.^27^

Fig. 2 illustrates the current–voltage characteristics of a solar cell circuit under two conditions: in the dark and under illumination.^27^ In the absence of illumination, when the electrons traverse from the anode to the cathode or when the circuit is subjected to reverse bias, no current will flow due to the significant energy barrier presented by the donor material. Only after overcoming this energy barrier, a minimal current will be observed. This behaviour is depicted by the dark curve. Conversely, when the solar radiation is absorbed by the donor material, the generation of charge carriers becomes more facile. As a result, a reverse current occurs, where electrons flow from the anode to the cathode. This reverse current, in the absence of an external voltage, is referred to as the short circuit current density (*J*_SC_). This phenomenon is represented by the hollow bubbled curve. Key performance parameters include the open-circuit voltage (*V*_OC_), defined by the difference between the quasi-Fermi level of electrons in TiO_2_ and the redox potential of the electrolyte:8
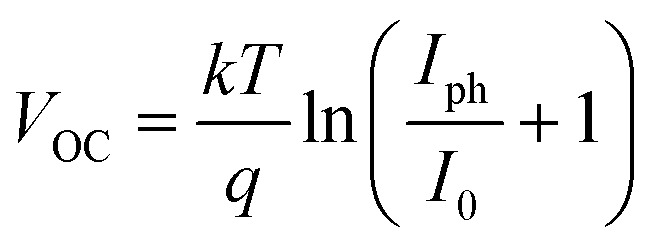


By applying a forward voltage to the solar cell, it is feasible to counteract the short circuit current, effectively compensating for its flow. This compensation process continues until a specific voltage is reached, at which point the current diminishes to zero. This specific voltage is termed the *V*_OC_. On the other hand, the open circuit voltage represents the maximum attainable voltage across the terminals of a solar cell when it is not connected to an external load. In this state, no external current is drawn from the cell, and it operates under zero current conditions. The short-circuit current (*I*_SC_) is the maximum current delivered when *V* = 0, and the fill factor (FF) is a measure of the shape of the current–voltage (*I*–*V*) curve, given by:9
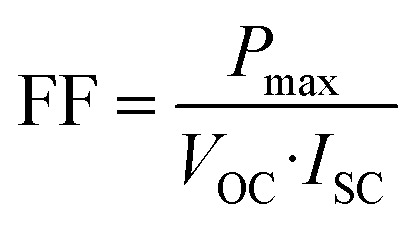
where *P*_max_ is the maximum power output. The power conversion efficiency (PCE) is then expressed as:10
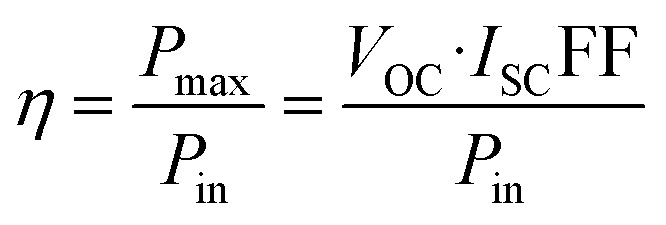
where *P*_in_ is the incident solar power.

This model facilitates a detailed understanding of DSSC performance and helps in optimizing parameters to enhance efficiency under varying operational conditions.

## 3. Importance of photoanodes in energy conversion

**Fig. 3:**
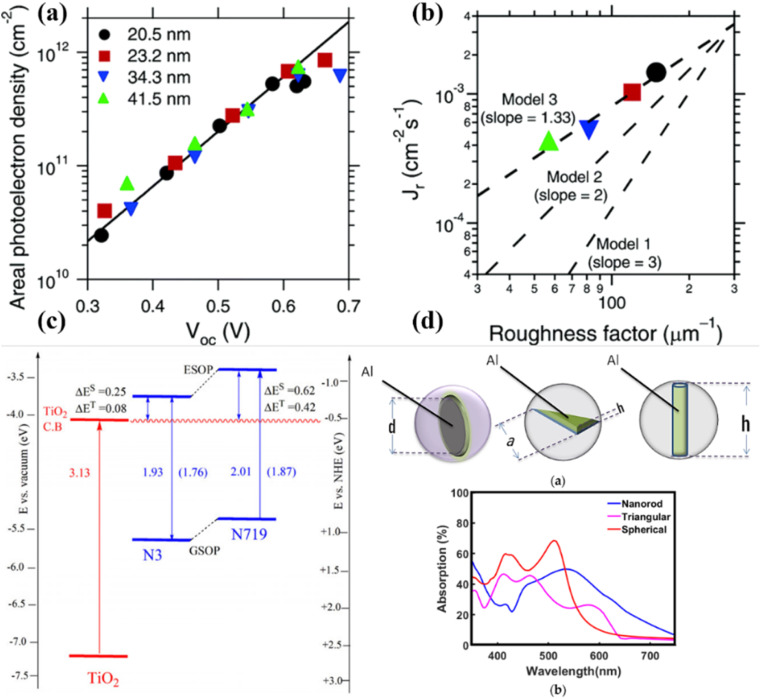
Important features of photoanodes that can affect the DSSC performance significantly (a and b) photoanodes surface area and roughness-dependent photocurrent density,^28^ (c) suitable bandgap alignment for better charge injection and transfer,^29^ and (d) photoanode morphology-dependent tuning of light absorption capability, causing enhanced performance.^30^

The photoanode plays a critical role in DSSCs by influencing photocurrent, photovoltage, and overall power conversion efficiency through its functions in dye absorption, electron injection, transport, and collection. Enhancing DSSC performance requires selecting suitable photoanode materials and optimizing their morphologies. An ideal photoanode must possess a high surface area to enable extensive dye adsorption, thereby improving light-harvesting capabilities (Fig. 3(a and b)).^28,31^ Efficient electron transport is crucial for the rapid injection and transfer of electrons to the external circuit, ensuring effective utilization of absorbed photon energy. Proper band gap alignment between the photoanode and sensitizer facilitates optimal energy transfer, while high resistance to photo corrosion ensures stability and durability under prolonged light exposure (Fig. 3(c)).^29,32^ Additionally, optimizing pore size is essential for efficient dye and electrolyte diffusion, enabling improved mass transport within the cell.^32^ The photoanode material should also demonstrate strong sunlight absorption or scattering capabilities (Fig. 3(d)) to enhance light utilization and conversion efficiency.^30^ Furthermore, establishing effective contact between the photoanode, dye molecules, and conducting substrate is vital for ensuring efficient charge transfer and electron utilization.^33^ This review focuses on TiO_2_ nanomaterials as photoanodes in DSSCs, highlighting their distinctive photovoltaic properties and key modifications to enhance performance. It provides a comprehensive overview of advancements in one-dimensional TiO_2_ morphologies and recent efforts to optimize their application as photoanode materials in DSSCs.

As shown in Fig. 3(a), Zhu *et al.*^28^ demonstrated the influence of TiO_2_ photoanode active surface area on charge transport and recombination. Fig. 3(a) shows a clear dependency of areal photoelectron density (*n*_oc_^*δ*^) on the *V*_OC_. The *n*_oc_^*δ*^ is directly proportional to the film's roughness factor, as evidenced by similar values between 0.3 and 0.6 V for films with varying roughness factors, indicating that photoinjected electrons primarily reside on the particle surfaces under open-circuit conditions. At *V*_OC_ > 0.6, a slight deviation from the exponential dependence occurs due to a band-edge shift caused by Helmholtz layer charging at the TiO_2_ particle/electrolyte interface.^26,34^ This results in a more rapid increase in *V*_OC_ with photoelectron density than predicted by exponential dependence. However, the normalized photoelectron density values remain consistent across samples with differing roughness factors.

In the same study, the recombination current density (*J*_r_) as a function of the roughness factor (*r*_f_) at *V*_OC_ = 0.55 V, with predictions based on three kinetic models. Model 1 predicts *J*_r_ ∝ (*r*_f_)^3^, assuming a constant recombination rate (*k*_1_) and two-electron interfacial charge transfer. Model 2 predicts *J*_r_ ∝ (*r*_f_)^2^, assuming *J*_r_ is first-order in photoelectron density and *k*_2_ is constant at fixed *V*_OC_. Model 3 predicts *J*_r_ ∝ (*r*_f_)^1^,^33^ with *J*_r_ first-order in photoelectron density and *k*_3_ scaling with the electron diffusion coefficient, which depends on photoelectron density. The findings highlight that photoelectron density at an open circuit is proportional to the film roughness factor, with deviations at higher *V*_OC_ attributed to band-edge shifts. Additionally, recombination current density predictions from kinetic models emphasize the dependence on the roughness factor, reflecting varying assumptions about recombination processes and rate constants.

Further, Angelis *et al.* summarized the dye/TiO_2_ excited state energy levels in Fig. 3(c), and the excited state oxidation potential (ESOP) was calculated as the difference between the ground state oxidation potential (GSOP) and the lowest vertical excitation energy (*E*_0–0_). The ESOP values align well with experimental data, especially when *E*_0–0_ is derived from S_0_ → S_1_ transitions.^29^ Also, the roughness factor directly influences the absorption coefficient and, simultaneously, the performance as well. As depicted in Fig. 3(d), the maximum absorption coefficient is observed in rod-like structures and is comparably high as compared to the disc and film-shaped photoanodes. Therefore, to design a photoanode for energy conversion, we must take care of proper band alignment, surface roughness, and structure for better light scattering and performance.

## 4. Typical photoanodes used in DSSC

Photoanode materials in DSSCs can be classified into several categories based on their composition and properties. Here are some common classifications of photoanode materials in DSSCs.

### 4.1 Metal oxides

**Fig. 4:**
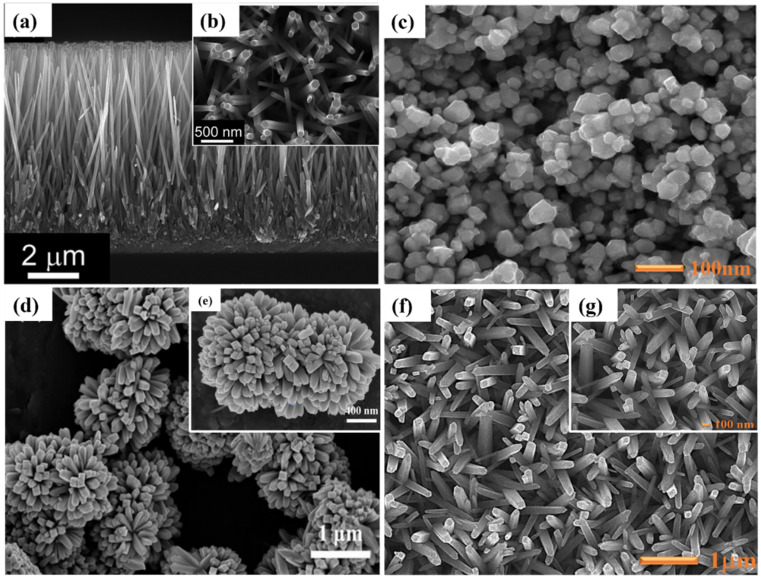
Typical nanostructured FESEM images of various metal oxides that were generally used as a photoanode in DSSC applications. (a and b) ZnO-nanorods,^35^ (c) Zn_2_SnO_4_-nanoparticles,^36^ (d and e) SnO_2_-nanoflower,^37^ and (f and g) TiO_2_-nanorods.^38^

Metal oxides are the most widely used class of photoanode materials in DSSCs. TiO_2_ is the most common and extensively studied metal oxide photoanode material due to its excellent stability, high electron mobility, and suitable band gap for efficient light absorption. Other metal oxides, such as SnO_2_, ZnO, Fe_2_O_3_, Nb_2_O_5_, SrTiO_3_, and Zn_2_SnO_4_, have also been explored as photoanode materials in DSSCs. The photoanode materials used in DSSCs are typically wide bandgap semiconductor materials. The performance of DSSCs is heavily influenced by the structural, morphological, and crystalline characteristics of these materials. The typical nanostructured morphology of these photoanode materials, which determines their electronic properties, can be observed in Fig. 4.^35–38^ The specific arrangement of atoms and the overall crystal structure significantly impact the light absorption, charge transport, and electron injection processes within the DSSC, ultimately influencing its overall performance.^39,40^

### 4.2 Nanomaterials

Nanomaterials such as nanoparticles, nanowires, nanotubes, and nanocomposites are widely employed as photoanodes in DSSCs due to their unique properties that enhance device performance. TiO_2_ nanoparticles, for example, offer a high surface area for increased dye adsorption and light harvesting, but their charge transport is limited by interparticle boundaries. In contrast, 1-D structures like TiO_2_ nanowires and ZnO nanorods provide direct electron transport pathways with reduced recombination, improving charge collection and electron diffusion lengths. TiO_2_ nanotubes combine a high surface area with unidirectional charge transport, leading to improved charge separation and reduced recombination, resulting in higher PCE. Additionally, nanocomposites such as graphene–TiO_2_ and ZnO–TiO_2_ synergistically enhance electron mobility and light absorption, further boosting PCE. These examples demonstrate how nanomaterials optimize the performance of DSSC photoanodes through tailored structural and electronic properties. The revised text has been incorporated to reflect this discussion.^41–43^

### 4.3 Composite materials

Composite photoanode materials, integrating multiple components, have been extensively studied to harness the synergistic advantages of each constituent. For instance, composites of metal oxides (*e.g.*, TiO_2_–ZnO) enhance charge transfer by combining the high electron mobility of ZnO with the superior dye adsorption and stability of TiO_2_. Similarly, incorporating carbon-based materials such as graphene improves electron transport and reduces recombination due to graphene's high conductivity and large surface area. Furthermore, the integration of organic semiconductors with metal oxides broadens light absorption into the visible range, enhancing photocurrent generation. These hybrid photoanodes improve overall device efficiency by simultaneously optimizing charge transport, light harvesting, and chemical stability, making them promising candidates for high-performance DSSCs.^44^

## 5. Optoelectronic suitability of TiO_2_ for DSSC application

The optoelectronic properties of TiO_2_ play a pivotal role in the performance and efficiency of DSSCs. TiO_2_, particularly in its anatase phase, has emerged as the most widely used photoanode material due to its unique combination of physical and chemical characteristics. These include a suitable bandgap, high electron mobility, excellent chemical stability, and a large surface area that enables effective dye loading, making it well-suited for light-harvesting applications in DSSCs. Firstly, the bandgap of anatase TiO_2_, approximately 3.2 eV, allows for efficient absorption of ultraviolet light, which constitutes a significant portion of the solar spectrum. Despite its wide bandgap limiting visible light absorption, various strategies, such as dye sensitization and bandgap tuning *via* doping, have been employed to enhance visible light capture. The intrinsic electronic structure of TiO_2_ ensures that photo-excited electrons are injected into the semiconductor's conduction band with minimal energy losses, leading to efficient charge separation.^45–47^

Furthermore, TiO_2_ exhibits favourable electron transport properties, with high electron mobility and a well-aligned conduction band edge that facilitates rapid electron transfer from the dye molecules to the photoanode. This minimizes recombination losses and ensures a high *V*_OC_ in DSSCs. The mesoporous morphology of TiO_2_ photoanodes is engineered to maximize the surface area for dye adsorption, thereby increasing light absorption and enhancing photocurrent generation. Surface modification of TiO_2_, such as using passivating agents or incorporating one-dimensional (1D) nanostructures like nanorods and nanotubes, further improves optoelectronic performance (detailed discussion in Section 6).^48,49^ 1D TiO_2_ nanostructures provide direct pathways for electron transport, reducing recombination and enhancing charge collection efficiency. Additionally, they enable improved light scattering and trapping, increasing the overall photon absorption within the device. Table 1 also combines all the most prominent optoelectronic features of TiO_2_ that are important to the DSSC application.

**Table 1:** Summary of typical optical and electrical properties of anatase, rutile, and brookite phase TiO_2_ photoanodes making it a suitable candidate for application in DSSC

Optoelectronic features	Anatase phase TiO_2_	Rutile phase TiO_2_	Brookite phase TiO_2_	Relevance to DSSC
Bandgap (*E*_g_)	∼3.2 eV	∼3.0 eV	3.1–3.4 eV	Anatase's higher bandgap favours efficient charge injection but limits visible light absorption. Rutile's narrower bandgap enhances visible light harvesting, while brookite's variable bandgap offers a balance for tailored applications
Conduction band edge (CB_edge_)	∼−4.2 eV (*vs.* vacuum level)	∼−4.0 eV (*vs.* vacuum level)	∼−4.1 to −4.4 eV (*vs.* vacuum level)	Anatase has a higher conduction band edge, beneficial for charge injection. Rutile's lower conduction band edge can reduce electron injection efficiency, while brookite provides a middle ground, potentially optimising both injection and transport
Electron mobility (*μ*)	0.1–4 cm^2^ V^−1^ s^−1^ (depending on the nano-structure and synthesis method)	∼0.1–1 cm^2^ V^−1^ s^−1^	∼0.5–2 cm^2^ V^−1^ s^−1^	Anatase has higher electron mobility, which reduces recombination losses. Rutile has relatively lower mobility, limiting performance, while brookite exhibits moderate mobility, which may be enhanced with proper engineering
Surface area (*A*)	∼50–200 m^2^ g^−1^ (mesoporous films)	∼20–50 m^2^ g^−1^ (lower surface area)	∼100–150 m^2^ g^−1^ (intermediate surface area)	Anatase's higher surface area allows for greater dye loading. Rutile, with a lower surface area, limits dye coverage, while brookite offers a compromise with moderate dye adsorption
Light scattering	Effective at 400–800 nm, enhanced in nanostructured forms	Moderate light scattering	Effective scattering, similar to anatase	Anatase and brookite nanostructures are particularly effective in enhancing light absorption. Rutile is less effective at scattering but can still be useful when structured appropriately
Chemical stability	Highly stable under UV and various pH conditions	Superior stability under harsh conditions	Moderately stable, less commonly studied in detail	All three phases are stable under typical DSSC conditions, but rutile is often preferred for harsh environments. Anatase remains the most widely used due to its balance of stability and performance
Nano structural versatility	Nanoparticles: 10–100 nm, nanorods: 500 nm-several μm	Nanoparticles: 50–150 nm	Nanoparticles: 20–80 nm	Anatase nanostructures (*e.g.*, nanorods) offer efficient electron pathways, enhancing performance. Brookite's structures need further investigation, while rutile structures are often used for enhanced light scattering in composites

The anatase phase of TiO_2_ is the most commonly utilized in DSSCs because it offers an optimal combination of high electron mobility, a suitable bandgap for efficient electron injection from dye molecules, and a large surface area that facilitates substantial dye loading. In contrast, the rutile phase is less frequently used on its own due to its lower electron mobility and limited surface area. However, rutile is often integrated into multi-phase systems to enhance light scattering and provide overall stability, making it a valuable component in composite structures. The brookite phase, while the least explored, presents promising intermediate properties that could be tailored for specific DSSC applications. It shows potential for performance optimization, especially when combined with anatase or rutile. Overall, this comparison highlights how each TiO_2_ phase can be leveraged or modified to improve DSSC performance, depending on the desired optoelectronic and structural characteristics.^50–53^

In summary, the optoelectronic properties of TiO_2_ are optimized through structural and surface engineering to achieve higher light-harvesting efficiency and charge carrier dynamics in DSSCs. These advancements underscore the significance of TiO_2_ in the continuous development of next-generation high-efficiency solar cells, with ongoing research focused on further tuning and enhancing its performance.

## 6. Nano-structured 1-D TiO_2_ photoanodes for DSSC

**Fig. 5:**
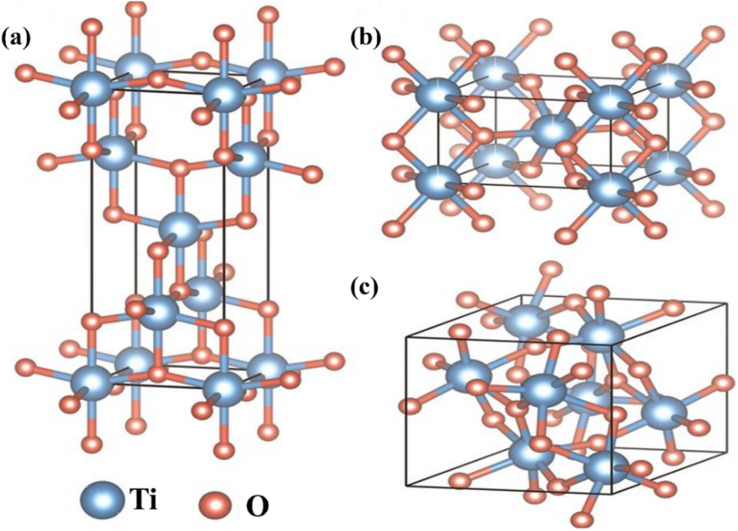
Typical ball-stick crystal structure of TiO_2_ polymorphs. (a) Anatase (tetragonal), (b) rutile (tetragonal), and (c) brookite (orthorhombic).^54^

It is worth noting that the classification of photoanode materials in DSSCs is not limited to these categories and continues to evolve as new materials and approaches are developed. TiO_2_ and ZnO are widely utilized materials in the fabrication of DSSCs, with TiO_2_ being the preferred choice due to its superior photovoltaic applicability compared to ZnO. Despite ZnO exhibiting higher electron mobility than TiO_2_, DSSCs employing TiO_2_ typically achieve higher efficiencies. This disparity in efficiency arises from ZnO's reduced dye adsorption capacity and its instability in acidic environments. TiO_2_ exists in four known polymorphs: rutile (tetragonal), anatase (tetragonal), brookite (orthorhombic), and TiO_2_ (B) (monoclinic), as illustrated in Fig. 5. Among these polymorphs, anatase is favoured over rutile for photovoltaic applications, despite rutile's greater stability and lower band gap. This preference stems from anatase's higher conduction band energy level, stronger light absorption characteristics, and lower electron–hole recombination rate. The synthesis of brookite TiO_2_ poses challenges, making it less explored and less applicable as a photoanode material in DSSCs.^54^

DSSCs utilizing anatase TiO_2_ have demonstrated efficiencies in the range of 12–14%. As a result, TiO_2_ is considered the most favourable choice as a photoanode material due to its cost-effectiveness, easy availability, good stability, non-toxicity, and suitable optical and electronic characteristics. Moreover, many stable sensitizers, known for their high light absorption capability, exhibit a favourable alignment of their lowest unoccupied molecular orbital (LUMO) energy level with the conduction band of TiO_2_. This alignment further enhances the performance of TiO_2_ photoanodes. However, the use of TiO_2_ as a photoanode material also presents challenges. Firstly, TiO_2_ has a relatively large band gap of 3.2 eV, which results in predominant absorption in the UV region.^55^ Secondly, TiO_2_ exhibits a low internal electron transport rate. To address these challenges and improve the functionality of TiO_2_ photoanodes, research efforts have focused on various factors such as increasing the surface area, enhancing light scattering effects, improving interface quality, achieving fast electron mobility, and enhancing charge collection abilities. Furthermore, the physical, chemical, and optical properties of TiO_2_ depend not only on its intrinsic electronic structure but also on its shape, size, porosity, pore size distribution, organization, and surface features. Exploring and optimizing these factors contribute to the overall performance enhancement of TiO_2_ in DSSCs.^56^

**Fig. 6:**
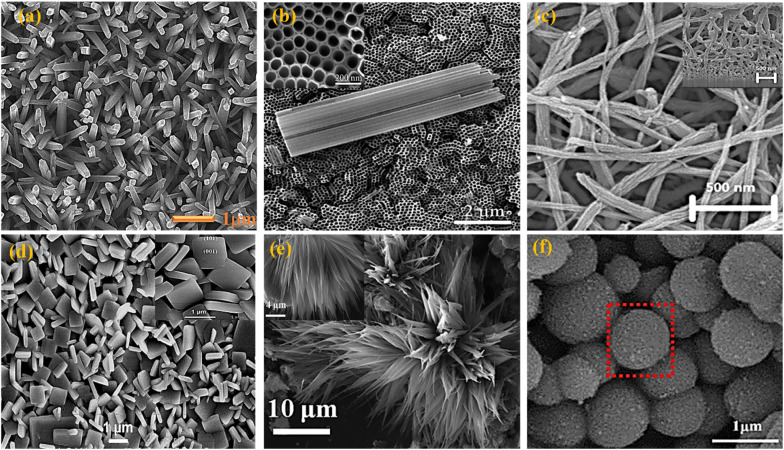
FESEM images of various TiO_2_ photoanodes including 1D, 2D and 3D structures explored for DSSC application. (a) Nanorods,^38^ (b) nanotubes,^57^ (c) nanowires,^58^ (d) nanosheets,^59^ (e) nanoflower,^60^ and (f) nanosphere.^61^

Achieving a higher dye pickup by the photoanode material leads to an increase in electron and current density generation. To enhance dye adsorption capabilities, various nanostructures of semiconductor mesoporous TiO_2_, including nanorods, nanotubes, nanowires, nanosheets, and other nanoarchitectures, have been extensively employed and investigated as shown in Fig. 6(a–f).

These nanostructures offer increased surface area, providing more binding sites for dye molecules and promoting efficient light absorption. In addition to maximizing surface area, enhancing electron mobility is crucial for improving photoanode performance. Defects present in TiO_2_, particularly at grain boundaries, can act as electron traps, hampering the collection of injected electrons from the semiconductor. Therefore, minimizing or eliminating defects is essential to ensure efficient electron collection and transport within the photoanode material. Surface modification techniques have also proven effective in improving the performance of TiO_2_ semiconductors. By modifying the surface properties, such as introducing surface coatings or functional groups, charge separation, electron mobility, and the recombination process can be significantly influenced. These surface modifications play a crucial role in facilitating efficient charge separation, reducing electron recombination, and improving overall photoanode performance in DSSCs.^57–61^

**Fig. 7:**
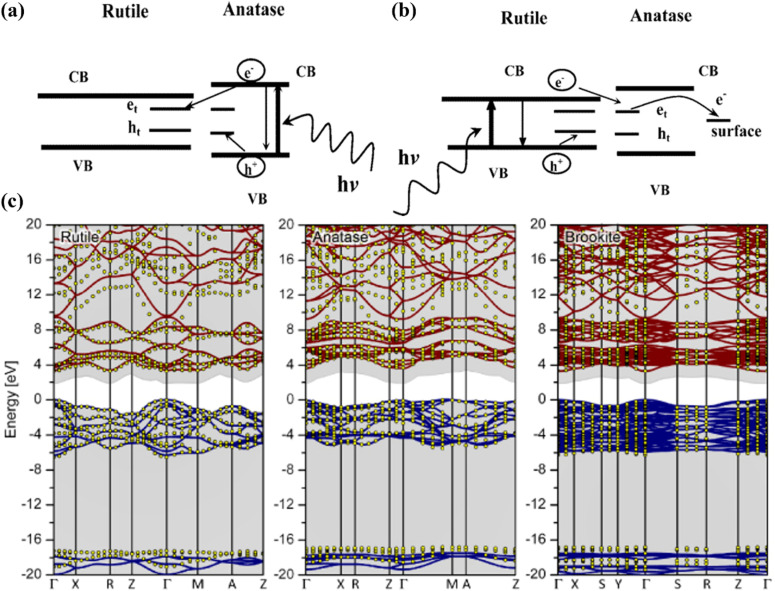
(a and b) Typical schematics of charge trapping process in two-phase TiO_2_ namely rutile and anatase, which illustrate that photogenerated charges in the anatase phase can be further trapped *via* injection in the rutile phase,^62^ (c) typical band structures and quasi-particle energies of different TiO_2_ phases (Rutile, Anatase, Brookite).^63^

Although 2D materials offer a high surface area for dye adsorption, 1D tubular structures like nanotubes and nanowires excel in charge transport and light scattering. Their elongated surface area promotes multi-path charge transport, reducing recombination losses and enhancing charge collection. Additionally, these structures provide better light scattering, improving light absorption across a broader spectrum. The 1D design enables directional charge transport along the *z*-axis, further optimizing photoanode performance. Thus, the choice of 1D tubular structures is justified by their balance of high surface area and efficient charge transport (Fig. 7).^64,65^

The optical and electronic properties of TiO_2_ are significantly influenced by its various morphologies. It has been observed that one-dimensional nanostructures such as nanowires, nanorods, and nanotubes exhibit superior charge transport properties compared to assemblies of TiO_2_ nanoparticles. However, these one-dimensional structures possess a smaller surface area than nanoparticle systems, resulting in reduced dye adsorption capability. To address this limitation, the introduction of dopants with enhanced dye adsorption capacities can improve the photovoltaic performance of one-dimensional nanostructures.^66^ This allows for better utilization of incident light and improved conversion of photons into charge carriers. On the other hand, nanoparticle assemblies can benefit from dopants that promote increased charge transfer processes. These dopants facilitate efficient electron transfer between the TiO_2_ nanoparticles, enhancing the overall conductivity and charge transport within the material. As a result, it becomes challenging to determine the precise contribution of absorption enhancement or electronic effects in doped TiO_2_ towards the improvement of its performance. Both factors, the increased absorption of light due to enhanced dye adsorption and the improved charge transfer properties resulting from dopants, play a crucial role in enhancing the overall photovoltaic performance of doped TiO_2_ materials.^67^

Nanotubes, nanowires, nanofibers, nanobelts, and nanoribbons are a few examples of one-dimensional nanostructures that have found extensive use in DSSCs. The ground-breaking research by Iijima *et al.* is largely responsible for the growing interest in these nanomaterials.^68^ For electron transport from the semiconductor layer to the conducting substrate, their orderly alignment provides an identifiable route. This has two effects: first, it slows down the rate of electron recombination, and second, it improves the solar cell's efficacy.

### 6.1. 1-D TiO_2_ nanowires

**Fig. 8:**
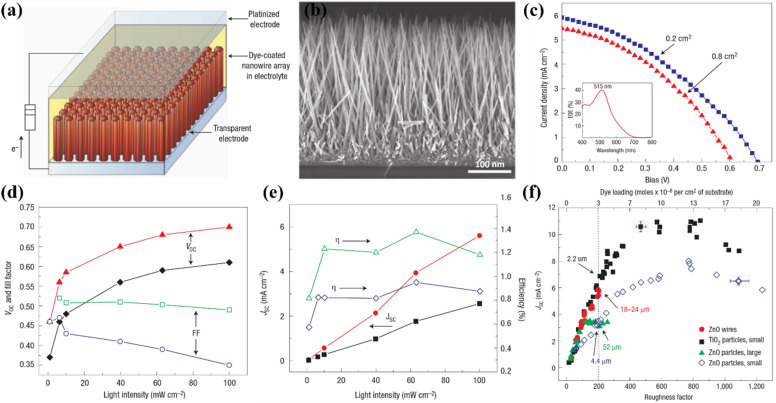
(a) Schematic DSSC fabricated using TiO_2_ nanowires as a photoanode, (b) typical FESEM image of cross-sectional view of TiO_2_ nanowires, (c) *J*–*V* analysis of respective DSSC with two different active area of 0.2 cm^2^ and 0.8 cm^2^ along with quantum efficiency curve shown inset, (d and e) trace of *V*_OC_, FF, *J*_SC_, and *η* against light irradiance, (f) comparative analysis of DSSC prepared using nanoparticles and nanowires with respect to roughness and dye-loading.^69^

In DSSCs, TiO_2_ nanowire (TNW) architectures are significant because they function as constrained conducting channels with long charge diffusion lengths that efficiently avoid charge recombination and promote optimal charge transport. They are now considered to be potential candidates for use in the manufacture of DSSC due to their special characteristics. When used as dye scaffolds, a dense array of long, thin nanowires can dramatically improve both dye absorption and carrier collecting efficiency. Additionally, nanowire photoanodes show superior compatibility with unconventional electrolytes such solid inorganic phases or polymer gels, which normally have greater recombination rates (Fig. 8).^69^

**Fig. 9:**
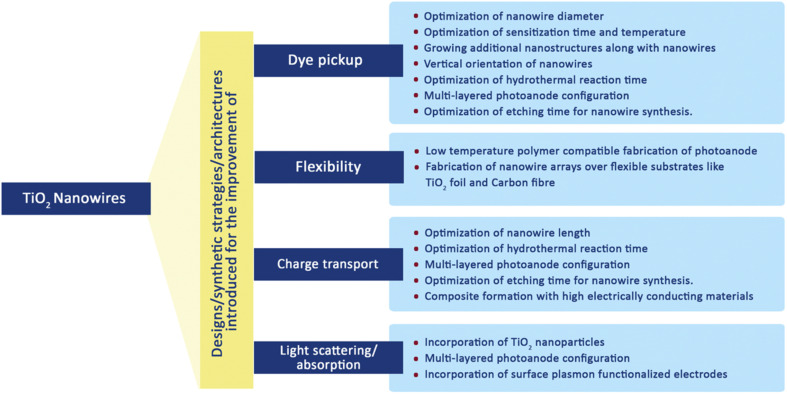
Various modifications made in TiO_2_ nanowires to improve the DSSCs performance.^70^

The electron–hole recombination time and electron collection time ratio of TNW-based DSSCs are exceptional, being around 150 times higher than those of nanoparticle-based solar cells. This substantial variation emphasises the higher collecting efficiency attained with nanowire arrays. The various initiatives made to improve the critical aspects affecting the overall efficiency of TNW-based DSSCs are summarised in Fig. 9.^70^

**Fig. 10:**
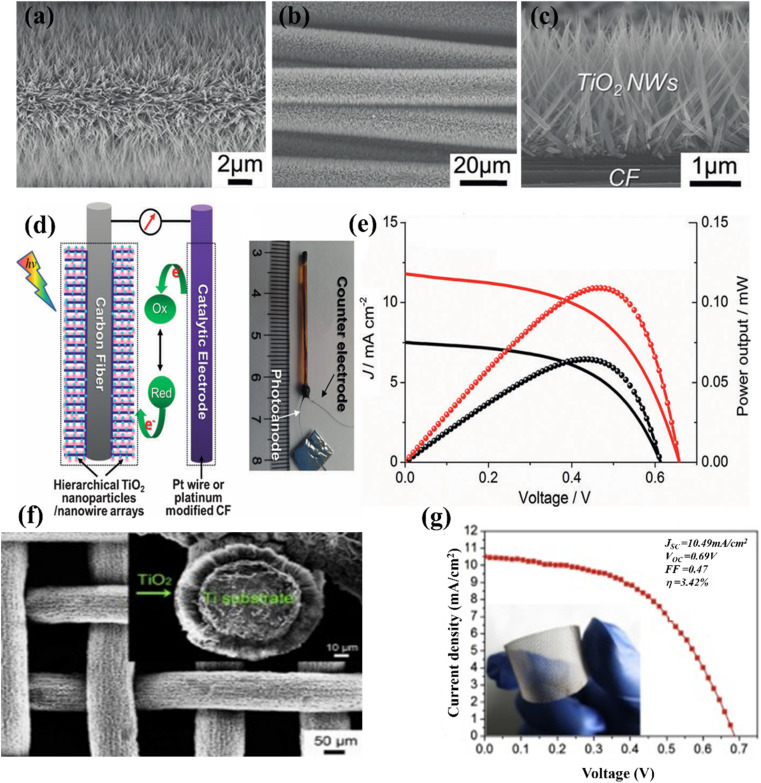
(a–c) FESEM images of hierarchical TiO_2_ nanowire arrays grown over the CF substrate at 190 °C for 80 minutes, (d and e) schematic of wire DSSC consisting TiO_2_ nanoparticle and nanowires and Pt-modified CF counter electrodes and consequent *J*–*V* analysis, (f and g) low-magnification FESEM images of the TiO_2_ NWAs on Ti mesh substrates and respective *J*–*V* performance.^72^

Feng *et al.* introduced a simple technique for producing single-crystalline rutile TNWs at low temperatures. The method involves a nonpolar solvent and a hydrophilic solid substrate interfacial reaction under hydrothermal conditions. Using this approach, they were able to grow vertically aligned nanowires with lengths of up to 5 μm on TCO glass substrates. When N719 dye was applied to the TNW arrays measuring 2–3 μm in length, the system achieved an efficiency of 5.02% under AM1.5 irradiation.^71^ Consequently, the implementation of low-temperature techniques for photoanode fabrication is compatible with polymer substrates, enabling enhanced flexibility in the process. A hydrothermal technique capable of precisely creating TNW arrays on a titanium mesh with an average diameter of 80 nm was presented by Liu *et al.* (2015). They investigated the influence of sensitization temperature and time on the performance of TNW arrays in DSSCs. They observed that higher sensitization temperatures enhanced dye infiltration into the internal regions of the TNW films, leading to improved photovoltaic performance. Under optimal conditions, they achieved an efficiency of 3.42% for a flexible DSSC, demonstrating effective dye coverage and reduced charge recombination (Fig. 10(f–g)).^72^ Additionally, in addition to attaining the intended dye uptake, their method also effectively reduced charge recombination problems.

**Fig. 11:**
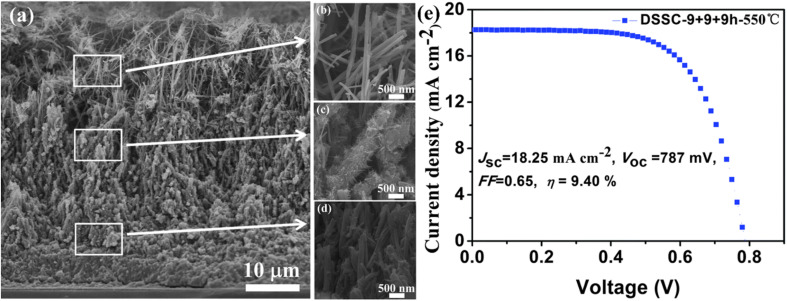
(a) Cross-sectional FESEM image of TiO_2_ nanowires grown on FTO substrate in 3 cycles of 9 h each, (b–d) FESEM images of top, intermediate, and bottom layers of grown nanowires, (e) *J*–*V* analysis of the DSSC prepared using these TiO_2_ nanowires having total thickness of 47 μm.^75^

In contrast, Bakshayesh *et al.* employed a corn-like nanowire structure fabricated through a surface tension stress mechanism, achieving a dual-layer photoanode comprising a bottom layer of anatase TiO_2_ nanoparticles and a top layer of corn-like TNWs. This design enhanced light scattering, dye sensitization, and charge carrier generation, resulting in a higher efficiency of 7.11%. The increased surface area of corn-like nanowires significantly improved dye uptake and *J*_SC_, while the nanoparticles further amplified light scattering, boosting overall device performance.^73^ Zha *et al.* focused on controlling the length of the nanowires, producing 6 μm long TNWs through an 8 hours synthesis process. This approach highlighted the critical role of microstructural properties in DSSC efficiency, with their system achieving a notable efficiency of 5.61%.^74^ Building on this, Wu *et al.* demonstrated the potential of vertically aligned TNWs with lengths adjustable between 15 and 55 μm for multi-layered photoanode configurations. By sensitizing these TNWs with N719 dye, they achieved an impressive efficiency of 9.40% (Fig. 11(a–e)), underscoring the importance of controlled nanowire alignment and length in enhancing light harvesting and charge transport in DSSCs.^75^

A unique DSSC created by Yen *et al.* successfully integrated plasmonic and antireflective properties. Their DSSC included a 3D TNW-AuNP plasmonic electrode made up of Au nanoparticles (NPs) attached to antireflective TNWs, which served as light-harvesting antennae. The absorption wavelength was increased from 520 nm to 575 nm as a result of the use of plasmonic functionalized electrodes (PFEs). This strategy intended to get beyond the drawbacks of standard DSSCs' small dye absorption ranges and poor dye absorption coefficients. TiCl_4_ treatment was used to further improve the TNW-Au-NP hybrid DSSCs' efficiency, leading to a notable improvement from 6.25% to 9.73%. It was shown that combining antireflective qualities with plasmonic effects was a successful way to boost the efficiency of DSSC.^76^

In their study, Wu *et al.* established a distinctive and inventive architecture by integrating various three-dimensional, hyperbranched titania nanostructures in a multi-stack configuration. The photoanode design comprised of three layers: a bottom layer composed of hyperbranched hierarchical tree-like titania nanowires, an intermediate layer featuring branched hierarchical rambutan-like titania hollow sub-micro-meter-sized spheres, and a top layer encompassing hyperbranched hierarchical urchin-like titania micro-meter-sized spheres. Each layer had a distinct function that improved the photoanode's overall performance. Efficient electron transport from 1-D nanowires to the FTO plate was made possible by the bottom layer. Due to the hollow-hole structure of the sub-micro-meter-sized macro-porous TiO_2_ spheres, the intermediate layer provided excellent light-trapping efficiency. On the other hand, more light might be scattered thanks to the higher layer of hyperbranched hierarchical TiO_2_ microspheres. The combination of these various nanoarchitectures produced an astounding photoanode efficiency of 11.01%, outperforming its TiO_2_ nanoparticle counterpart appreciably.^77^ The creation of outstanding performance photoelectrochemical devices for a variety of applications is greatly enhanced by this research. The previous discussion highlighted the substantial endeavors that were undertaken to enhance the performance of nanowire-based DSSCs, and a summary of these efforts is provided in Table 2.

**Table 2:** Comparative analysis of DSSCs fabricated *via* TiO_2_ nanowire photoanodes and their performance

Photoanode type	Dye	Electrolyte	PCE	Ref.
Vertically aligned single-crystalline TiO_2_ nanowire arrays by nonpolar solvent/hydrophilic solid substrate interfacial reaction under hydrothermal conditions	N719	MPN-100 (Solaronix, Inc., Switzerland) containing tri-iodide in methoxy–propionitrile	5.02%	71
Double-layered photoanode having corn-like TiO_2_ nanowires prepared by a surface tension stress mechanism	N719	Dimethylpropylimidazolium iodide, LiI, I_2_, and 4-*tert*-butylpyridine in acetonitrile	7.11%	73
Thornbush-like TiO_2_ nanowires (TBWs) prepared by a facile single-step hydrothermal method	N719	Dimethylpropylimidazolium iodide, LiI, I_2_, and 4-*tert*-butylpyridine in acetonitrile	6.7%	78
Double-sided brush shaped (DSBS) TiO_2_ nanoarchitecture consisting of highly ordered TiO_2_ nanowires aligned around an annealed TiO_2_ nanoparticle layer was prepared by a hydrothermal method	N719	1-Butyl-3-methyl imidazolium iodide, I_2_, guanidinium thiocyanate, and 4-*tert*-butylpyridine in a mixture of acetonitrile and valeronitrile	5.61%	74
Vertically aligned anatase TiO_2_ nanowires on FTO glass with a tunable length in the range of 15–55 mm for multilayered configuration of the photoanode by a hydrothermal method	N719	I^−^/I_3_^−^ redox electrolyte	9.40%	75
Photoelectrode with multi-stacked layers having integrated functions	N719	1-Methyl-3-propylimidazolium iodide (PMII), LiI guanidinium thiocyanate, I_2_, and *tert*-butylpyridine in acetonitrile and valeronitrile	11.01%	77
Vertically aligned rutile TiO_2_ nanowire arrays (NWAs) by a single-step solvothermal method without using any surfactant or template	C106	DMII, LiI, I_2_, TBP, and GNCS in the mixture of acetonitrile and valeronitrile	8.9%	79
3D TNW-AuNP plasmonic electrode prepared by hydrothermal and sputtering techniques	N719	I_2_, LiI, DMPII, and TBP in acetonitrile	9.73%	76
Rough surface rutile TiO_2_ nanowire array prepared by a hydrothermal method and prolonged etching. An additional light-scattering layer of TiO_2_ particles was also employed	C109	1,3-Dimethylimidazolium, lithium iodide, iodine, *tert*-butylpyridine, and guanidinium thiocyanate in acetonitrile and valeronitrile	9.39%	80

### 6.2. 1-D TiO_2_ nanorods

**Fig. 12:**
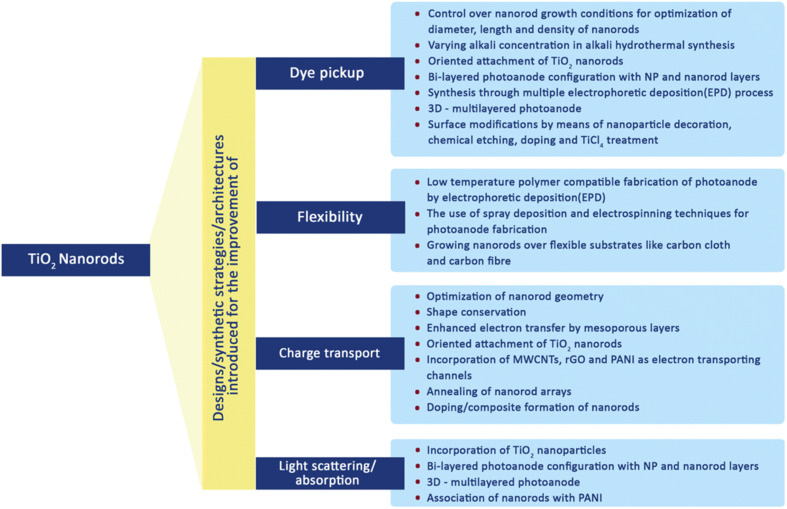
Various modifications made in TiO_2_ nanorods to improve the DSSCs performance.^70^

In order to improve the efficiency of DSSCs through the use of their special 1-D nanoscale properties, nanorods (NRs) have been introduced into the fabrication process of DSSCs. Due to their unique geometry, these TNRs have benefits in enhancing effective electron transport. The DSSCs can perform better overall by integrating TNRs because they can lessen the ohmic loss that ordinarily happens during the electron transfer process through the mesoporous titania layer. Researchers have looked into numerous processes for creating TNRs exclusively for DSSC applications in order to acquire the needed characteristics. Additionally, as illustrated in Fig. 12, they have worked very hard to change the morphology and surface characteristics of these nanorods.^70^ These efforts include the development of novel synthesis techniques that allow TNRs to be customized to match the unique needs of DSSCs and further increase their efficacy.

In order to exert control over the size and diameter of the nanorods, Jiu *et al.* in 2006 successfully synthesised single-crystalline anatase TNRs using a surfactant-assisted hydrothermal method. The resultant nanorods had a length that ranged from 100 to 300 nm and a diameter of 20 to 30 nm. The incorporation of these TNRs into DSSCs allowed the researchers to obtain a noteworthy efficiency of 7.29%.^81^ Because it permitted for improved electron transport qualities and more effective light absorption, the ability to adjust the size and diameter of the nanorods played a vital part in their efficacy within the DSSCs and eventually contributed to the better overall efficiency of the DSSC. This work by Jiu *et al.* opened up new opportunities for further developing solar cell technology by demonstrating the potential of using TNRs in DSSC photoanodes. De Marco *et al.* achieved a PCE of 7.9%, which represents a significant improvement in the performance of DSSCs. The utilization of TNRs, which were made using a single-step solvothermal method, allowed this substantial improvement. The produced anatase TNRs were afterward turned into a screen printable paste, making it simple to apply to DSSCs.^82^

**Fig. 13:**
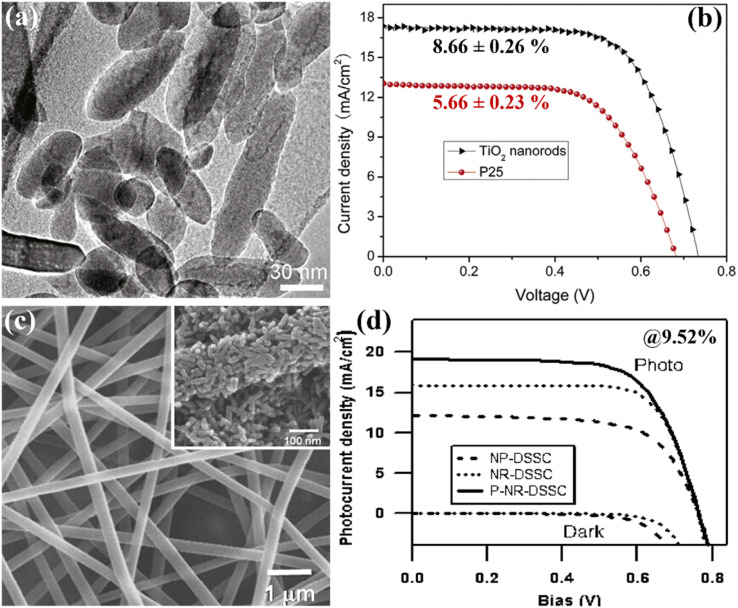
(a and b) High resolution TEM image of single crystalline anatase TiO_2_ nanorods and the respective DSSC performance with 8.66% PCE,^84^ (c and d) typical SEM image of electro-spun TiO_2_–PVAs nanofibers with heat treated @400 °C SEM image in inset and their consequent performance with PCE of 9.52%.^85^

Zhang *et al.* in their work suggested a unique method for fabricating anatase TNRs with controllable dimensions and shapes. They were able to create long, single-crystalline TNRs with reduced grain boundaries that improved charge collecting by using an orientated attachment technique. These long, skinny NRs of average thickness of 16 ± 0.5 μm allowed DSSCs to operate at an amazing 8.87% efficiency.^83^ Parallelly, Liu *et al.* created a solvothermal technique to create single-crystalline anatase TNRs using tetrabutylammonium hydroxide (TBAH) as a morphology-controlling agent. The DSSCs created with the help of these nanorods with thicknesses varying from 6.2 to 14.8 μm had a noteworthy PCE of 8.66% (Fig. 13(a and b)).^84^ A notable development in DSSCs was made in 2009 by Lee *et al.*, who attained a remarkable efficiency of 9.52%, as shown in Fig. 13(c and d). They used a hybrid of electrospinning and sol–gel methods to create TNRs from a solution of polyvinyl acetate and titanium *n*-prop-oxide in dimethyl formamide. They compared the performance of DSSCs based on nanoparticles and nanorods of average diameters of ∼15 nm and lengths of 60–100 nm. Through their research, they noticed that the pore volume of the TNR-DSSCs was double that of the nanoparticle-DSSCs. When compared to nanoparticle-based DSSCs with comparable masses of TiO_2_, the TNR-based DSSCs had almost 2.5 times as much surface area accessible as sensitizers.^85^ Additionally, nanorods showed an 8-times prolonged electron–hole recombination time than nanoparticle DSSCs, indicating greater charge separation and retention. Multi-walled carbon nanotubes (MWCNTs) were incorporated into TNRs with an optimum thickness of 14.3 ± 0.3 μm using the electrospinning method in 2013, which Yang *et al.* dubbed “electron transporting superhighways”. The inventive addition of MWCNTs improved the DSSCs' efficiency even more, increasing it to an astonishing 10.24%.^86^

There was subsequently a rise in interest in fusing other nanomaterials with TiO_2_ nano-morphologies. One such notable photoanode material has a double-layered structure with ZnO nanoflowers embedded in TiO_2_ as the bottom layer and TNRs as the top layer. Chen *et al.* successfully achieved a PCE of 8.01% by using optimized 10 μm thick ZNFs@TNPs + TNR integrated photoanodes. This innovation demonstrates the investigation of innovative nanomaterial combinations to improve photoanode performance for possible use in photovoltaic devices.^87^ Subramaniam *et al.* succeeded in overlaying the TNR surface with a layer of reduced graphene oxide (rGO) to increase the nanorods' charge collection efficiency. An outstanding PCE of 4.54% was achieved because of the addition of a 2 wt% rGO-loaded nanocomposite, which greatly increased photoconversion efficiency.^88^ Later studies by Tang and team revealed a connection between the efficacy and ability of dye loading on TNRs and their aspect ratios. They discovered that the dye loading efficiency and capacity both rose when the NRs' aspect ratios did. They managed the response time during the preparation procedure to produce nanorods with various aspect ratios.^89^ Significant results from numerous investigations on TNR-based DSSCs are compiled in Table 3.

**Table 3:** Comparative analysis of DSSCs fabricated *via* TiO_2_ nanorod photoanodes and their performance

Photoanode type	Dye	Electrolyte	PCE	Ref.
TiO_2_ single crystalline anatase nanorods prepared by a surfactant-assisted hydrothermal method	N719	LiI, 1,2-dimethyl-3-*n*-propylimidazolium iodide (DMPII), I_2_, and 4-*tert*-butylpyridine (TBP) in methoxyacetonitride	7.06%	90
Highly crystalline TiO_2_ nanorods synthesized by a hydrothermal process in a cetyltrimethylammonium bromide surfactant solution	N719	LiI, 1,2-dimethyl-3-*n*-propylimidazolium iodide (DMPII), I_2_, and 4-*tert*-butylpyridine (TBP) in methoxyacetonitride	7.29%	81
TiO_2_ nanorod based photoelectrodes prepared by a combination of sol–gel chemistry and electrospinning	N719	1-Butyl-3-methylimidazolium iodide, iodine, guanidinium thiocyanate, and 4-*tert*-butylpyridine in acetonitrile/valeronitrile	9.52%	85
TiO_2_ anatase nanorods prepared by a simple one-step solvothermal method	N719	LiI, I_2_, 1-methyl-3-propylimidazolium iodide, and *tert*-butylpyridine in dried acetonitrile	7.9%	82
1 : 1 TiO_2_ NR–NP composites prepared by a hydrothermal technique with a hydrogen titanate nanorod precursor	N719	LiI, I_2_ and 4-*t*-butylpyridine in acetonitrile	8.61%	91
Ultraporous anatase TiO_2_ nanorods fabricated by a simple microemulsion electrospinning approach	N719	I_2_, LiI, 1-methyl-3-propylimidazolium iodide (PMII), guanidinium thiocyanate, and *tert* butylpyridine in a mixture of acetonitrile/valeronitrile	8.53%	92
TiO_2_ microspheres assembled by single crystalline rutile TiO_2_ nanorods were synthesized by one-pot solvothermal treatment	N719	I_2_, LiI, 1-methyl-3-propylimidazolium iodide (PMII), guanidinium thiocyanate, and *tert*-butylpyridine in a mixture of acetonitrile/valeronitrile	8.22%	93
MWCNTs are introduced into TiO_2_ nanorods by electrospinning	N719	I_2_, LiI, 1-methyl-3-propylimidazolium iodide (PMII) and 4-*tert*-butylpyridine in a mixture of acetonitrile/valeronitrile	10.24%	86
Single crystal-like anatase TiO_2_ nanorods with a specific growth direction are prepared by a hydrothermal method	Z907	1,3-Dimethylimidazolium iodide, LiI, and I_2_ in a mixture of acetonitrile and valeronitrile	8.87%	83
Single-crystalline anatase TiO_2_ nanorods were prepared by a solvothermal method	N719	LiI, I_2_, dimethylpropylimidazolium iodide (DMPImI) and *tert*-butylpyridine in dry acetonitrile	8.66%	84
Monodispersed TiO_2_ nanorods were prepared using a simple solvothermal process	N719	Lithium iodide, iodine, 4-*tert*-butylpyridine and 1,2-dimethyl-3-propylimidazolium iodide was dissolved in acetonitrile	9.21%	94
Single-crystalline anatase TiO_2_ nanorods with a high aspect ratio	N719	LiI, I_2_, dimethylpropylimidazolium iodide (DMPImI) and *tert*-butylpyridine in a dry mixed solution	7.51%	89
Double layered photoanode having an overlayer of a TiO_2_ NR array and underlayer of a TiO_2_ embedded ZnO nanoflower array by a sol–gel method	N719	LiI, I_2_ and LiClO_4_ in acetonitrile	8.01%	87
Rutile TiO_2_ nanorods incorporated with α alumina were developed on an FTO surface *via* a hydrothermal route	N719	KI, I_2_ and 4-*tert*-butyl pyridine	6.5%	95

### 6.3. 1-D TiO_2_ nanotubes

**Fig. 14:**
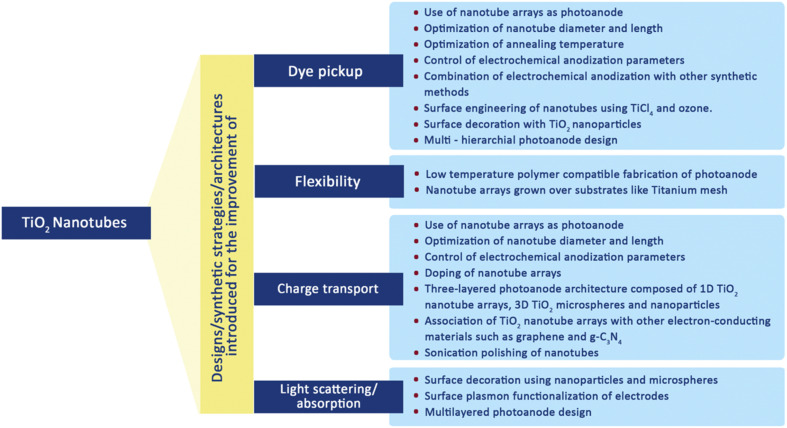
Various modifications were made to the TiO_2_ nanotubes to improve the DSSC's performance.^70^

TiO_2_ nanotubes (TNTs), which have a distinctive hollow cavity architecture and a much greater active surface area, were the subject of research. TNTs generally showing exceptional abilities in terms of increased absorption capacity and quick electron transport, making them particularly appropriate for use in DSSCs. TiO_2_ meso-structures can successfully avoid the emergence of electron traps by using nanotube arrays with small spaces between them. The diffusion length, which measures the distance an electron may travel inside a tube before engaging in recombination, is significantly improved as a result of this estimated strategy. According to calculations, a nanotube cell's diffusion length is approximately 100 μm.^96^ In order to increase the surface area while also lowering the likelihood of recombination, the size of the nanotubes can be extended up to this threshold. TNTs have some disadvantages, chief among them being the high cost of production and the time-consuming preparatory procedures needed. The morphology and crystalline structure of the TNTs used in DSSCs significantly impact their efficiency. Contrary to popular belief, it has been found that decreasing tube diameter has a greater positive influence on efficiency improvement than increasing tube length. The annealing temperature that the nanotubes go through also affects how much dye is put into them. Harvesting efficiency is another important aspect that affects performance, and it can be improved by making changes to the tube surfaces that reduce recombination losses.^97^ The efforts made to improve the efficiency of TNT-based DSSCs are shown in Fig. 14.^70^

**Fig. 15:**
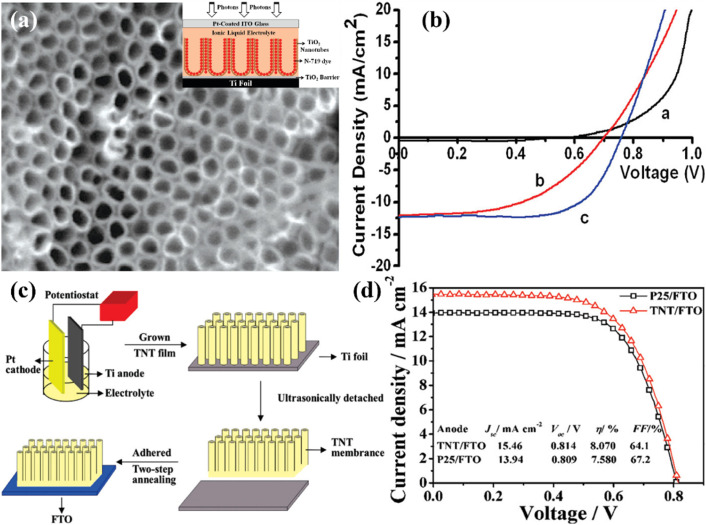
(a) FESEM top-view of highly ordered TiO_2_ nanorod arrays and inset image is a schematic illustration of fabricated DSSC, (b) *J*–*V* characteristics of the prepared device (black: under dark light, red: without TiCl_4_ treated anatase TiO_2_ nanotube array, and blue: TiCl_4_ treated TiO_2_ based DSSC),^98^ (c) schematic procedure of fabricating crystallized TiO_2_ nanotubes on FTO substrate, (d) Comparative *J*–*V* analysis using the same thickness (20.8 μm) of TiO_2_ nanotubes/FTO film and P25/FTO film under AM 1.5G illumination (100 mW cm^−2^).^99^

A study by Wang *et al.* showed that a 14 μm elongated TNT array in DSSCs can be made more effective by being treated with TiCl_4_ and ozone. They attained an efficiency of 7.37% by using this surface engineering strategy, as shown in Fig. 15(a and b).^98^ Similar to this, Lei *et al.* used an optimized 20.8 μm length of 1-D TNT arrays made by anodization to reach an efficiency of 8.07% (Fig. 15(c and d)).^99^ Another study by Hun Park *et al.* revealed that after undergoing treatment with TiCl_4_, TNTs longer than 15 μm that were transplanted onto an FTO plate had a PCE of 5.36%.^100^ Researchers looked into adding dopants to TNT arrays alongside surface engineering to increase performance. They intended to obtain an ideal alignment between the LUMO of dye molecules and the conduction band of TNTs in order to facilitate effective electron transfer. This alignment was predicted to improve electron injection and decrease electron recombination, improving overall efficiency. Numerous research teams investigated other strategies for doping to improve TNT arrays in DSSCs, such as surface decorating with nanoparticles and microspheres. It was shown by Roy *et al.* that treating TNTs with TiCl_4_ caused the TNT surface to be decorated with TiO_2_ nanocrystals. The overall efficiency was raised as a result of the much-increased dye absorption provided by the surface ornamentation.^101^ He *et al.* used microwave-created TiO_2_ microspheres to decorate a TiO_2_ NTA with a total thickness of 17.4 μm. This method can potentially improve the functionality of DSSCs because adding these microspheres led to an efficiency of 7.24%.^102^

**Fig. 16:**
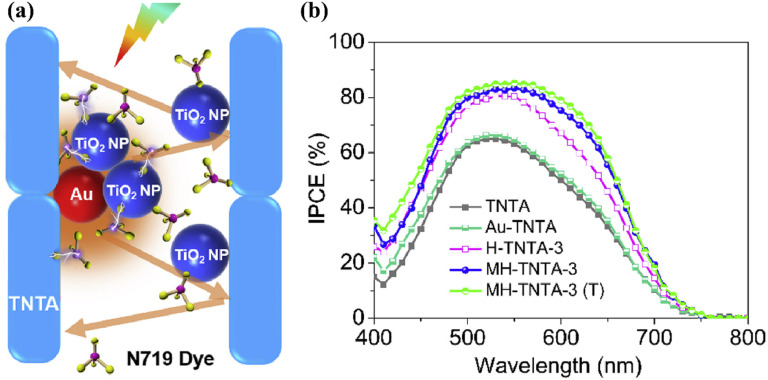
(a) Schematic illustration of Au-NPs induced plasmonic effect and enhanced light-trapping effect in the MH-TNTA film, (b) consequent IPCE spectra of DSSCs based on various photoanodes.^103^

Fu *et al.* accomplished a substantial development by creating an intriguing multi-hierarchical photoanode with a cigar-shaped Au/TiO_2_ NTR/TiO_2_ nanoparticle structure in 2019. This photoanode with a thickness of 15.2 μm had an amazing PCE of 8.93% (Fig. 16(a and b)).^103^ A novel vacuum-assisted colloid filling method was used to develop this one-of-a-kind multi-hierarchical design, which led to a photoanode with four times better charge transfer and 3.2 times greater dye intake than standard TNT array-based photoanodes. A hybrid photoanode that combines ZnO nanorods with TNTs having 90 nm inner diameter and ∼4 μm length was proposed by Bozkurt *et al.* in a different work.^104^ This hybrid photoanode was created using a two-step synthetic procedure that started with the anodic oxidation of TNTs and ended with the hydrothermal layering of ZnO nanorods on top of the TNTs. This hybrid strategy shows promise in improving photoanode effectiveness for prospective use in DSSC. Comparing the hybrid design to the conventional TNT photoanode, the PCE was increased by around a factor of two.

A ground-breaking 3-layered photoanode structure of 1D TNT-arrays, 3D TiO_2_ microspheres, and 0-D nanoparticles was developed by Wu *et al.* in 2014. This creative design displayed outstanding inherent characteristics, such as a high dye loading capacity, effective light scattering performance, and improved charge collection ability. These beneficial characteristics allowed this innovative structure to achieve an unparalleled 9.10% PCE.^105^ In order to further investigate and improve their performance, multiple other research groups have imitated this technique by producing tri-layered photoanodes with a variety of changes. In general, many techniques for altering TNTs have been employed, which includes (a) gluing on light-harvesting molecules for sensitization of nanotubes, (b) doping with multiple components, (c) band gap tuning, (d) developing electronic heterojunctions, and (e) furnishing with additional semiconductor particles.^106^ An overview of the many uses of tubular TiO_2_ nanoparticles in DSSCs is given in Table 4.

**Table 4:** Comparative analysis of DSSCs fabricated *via* TiO_2_ nanotubes photoanodes and their performance

Photoanode type	Dye	Electrolyte	PCE	Ref.
Titania nanotubes were synthesized using molecular assemblies	N3	0.03 M iodine in 0.3 M lithium iodide in 3-methyl-2-oxazolidinone (NMO)/acetonitrile	4.88%	107
Highly ordered TiO_2_ nanotube arrays were fabricated by electrochemical anodization followed by surface engineering using TiCl_4_ and O_2_ plasma	N719	BMIM-I, I_2_, TBP, and GTC in acetonitrile/valeronitrile	7.37%	98
Highly ordered one-dimensional TiO_2_ nanotube arrays were prepared by anodization of pieces of Ti foil	N719	I_2_, 1-methyl-3-propylimidazolium iodide (PMII), guanidinium thiocyanate, and *tert*-butylpyridine in acetonitrile and valeronitrile	8.07%	99
Layer-by-layer assembly of self-standing titania nanotube arrays and NPs were prepared by anodization	Ru535-bisTBA	1,2-Dimethyl-3-propyl imidazolium iodide, LiI, I_2_, and 4-*tert*-butylpyridine in acetonitrile	8.80%	108
TNTAs were prepared by anodization of pieces of Ti foil in an aqueous solution containing hydrofluoric acid and TiO_2_ scattering microspheres were prepared *via* a microwave solvothermal process	N719	I_2_, 1-methyl-3-propylimidazolium iodide (PMII), guanidinium thiocyanate, and *tert*-butylpyridine in a solution of valeronitrile and acetonitrile	7.24%	102
Double layered photoanode with a 1D NT underlayer and 3D hierarchical upper layer was prepared by a hydrothermal method and then incorporated with hydrothermally prepared TiO_2_ NPs	N719	1-Methyl-3-propylimidazo-lium iodide (PMII), guanidinium thiocyanate, I_2_, LiI, and *tert*-butylpyridine dissolved in acetonitrile and valeronitrile	9.10%	105
TiO_2_ NTAs prepared by anodization were transplanted on to a P25 coated FTO plate and then flower like TiO_2_ prepared hydrothermally was deposited over a P25/NTA film	N719	LiI, I_2_, 4-*tert*-butylpyridine and 1, 2-dimethyl-3-propylimidazolium iodide (DMPII) in dry acetonitrile	6.48%	109
“Cigar-like” Au/TiO_2_-nanotube-array/TiO_2_-nanoparticle multi-hierarchical photoanode through a novel vacuum-assisted colloid-filling approach	N719	LiI, I_2_, 1-methyl-3-hexylimidazolium iodide (HMII), *N*-methylbenzimidazole (NMB) and 4-*tert*-butylpyridine in 3-ethoxypropionitrile	8.93%	103
Tri-layered photoanode consisting of single crystal hollow TiO_2_ nanoparticles (HTNPs), sub-micro hollow TiO_2_ mesospheres (SHTMSs) and hierarchical TiO_2_ microspheres (HTMSs)	N719	1-Methyl-3-propylimidazo-lium iodide (PMII), guanidinium thiocyanate, I_2_, LiI, and *tert*-butylpyridine dissolved in acetonitrile and valeronitrile	9.24%	110
Open ended TiO_2_ nanotube array photoanode was prepared by fast removal of bottom caps by mechanical ball milling	N719	LiI, I_2_, 1-methyl-3- hexylimidazolium iodide (HMII), *N*-methylbenzimidazole (NMB), and 4-*tert*-butylpyridine in 3-methoxypropionitrile	7.7%	111

### 6.4 Engineered TiO_2_ photoanodes for DSSC

TiO_2_ photoanodes are critical to the performance of DSSCs, providing a high surface area for dye adsorption, efficient electron transport, and effective charge separation. The material's wide bandgap, high electron mobility, and chemical stability make it well-suited for this role. However, optimizing the crystalline phase, morphology, and surface properties of TiO_2_ is essential to enhance light harvesting, reduce recombination losses, and maximize efficiency. This section explores the engineered optoelectronic features of TiO_2_ photoanodes and their impact on DSSC performance.

#### 6.4.1. Doping

**Fig. 17:**
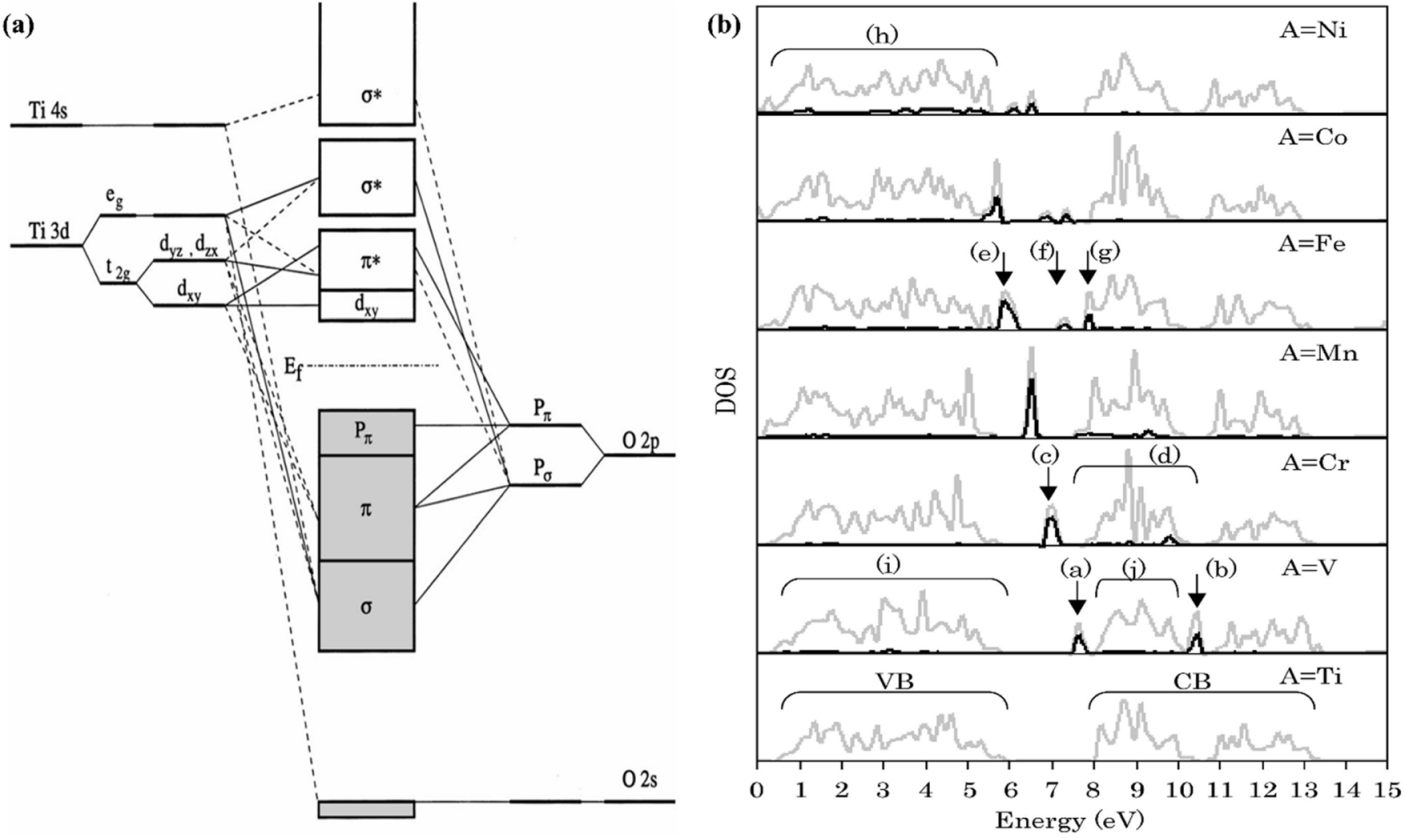
(a) Detailed molecular orbital bonding diagram of TiO_2_ composed of the components extracted from the DOS spectra,^112^ (b) density of states (DOS) of Ti_1−*x*_A_*x*_O_2_ doped *via* various metals (A: V, Cr, Mn, Fe, Co, Ni) where grey lines and black lines representing total DOS and dopant DOS respectively.^113^

Doped TiO_2_ has become crucial for enhancing the efficiency and performance of DSSCs. Incorporating various doping elements, such as transition metals and carbon, has been extensively studied to optimize the electronic properties of TiO_2_, resulting in improved charge transfer and enhanced light absorption. Doping in the TiO_2_ lattice can effectively modify its electronic properties through the intentional introduction of impurities. By incorporating dopants into the TiO_2_ structure, the number of free charge carriers and the overall conductivity of the material can be increased. This phenomenon arises from the inherent presence of defects within the TiO_2_ lattice, which directly influence the electronic structure and the occurrence of trap states within the material (Fig. 17(a)).^62,63^ During the doping process, either the Ti^4+^ cations or the O^2−^ anions can be substituted with other atoms. Consequently, the replacement of Ti^4+^ cations or O^2−^ anions leads to alterations in the conduction band (CB) and valence band (VB) structures, respectively. The CB energy levels are primarily formed by the orbitals of Ti^4+^ ions, while the VB energy levels predominantly consist of the O^2−^ 2p orbitals (Fig. 17(b)).^112,113^

Doping of TiO_2_ nanoparticles has been observed to have a significant effect on their growth rate, resulting in the formation of smaller particles with a greater surface area compared to undoped TiO_2_. This inhibition of growth can be attributed to the presence of dopant atoms within the TiO_2_ lattice, which introduce defects and alter the crystal structure, thereby hindering the crystal growth process. The increased surface area of doped TiO_2_ nanoparticles offers several advantages. Firstly, it enhances the adsorption capacity of dyes onto the TiO_2_ surface, leading to improved performance in applications such as DSSCs. The larger surface area provides more active sites for the dye molecules to attach, promoting efficient light absorption and conversion.^114^

Recently, Ju *et al.* demonstrated that doping TiO_2_ with transition metals (Cu and Zr) *via* a sol–gel method enhances the energy conversion efficiency of DSSCs. The doping extends light absorption into the visible region, improving electron generation and conductivity. Further, EIS and IMPS/IMVS analysis show reduced electron-transfer resistance and enhanced electron mobility, speed, and lifetime. As a result, Cu, Zr doping increases DSSC efficiency by 3.28%.^115^ TiO_2_ mesoporous structures were doped with indium (In) using a sol–gel method to enhance their performance as photoanodes in DSSCs, which was explored by Ayaz and the group. In-doping increased carrier concentration (1.1 × 10^17^ cm^−3^), conductivity, and charge transfer properties, as confirmed by Mott–Schottky and EIS, which showed reduced charge transfer resistance (33 Ω cm^2^). The TiO_2_ : In-based DSSC achieved an 8.62% efficiency, a 55% improvement over non-doped TiO_2_.^116^ In another report, Sharif *et al.* synthesized Ag-doped TiO_2_ nanomaterials (1–4% Ag) using a green-modified solvothermal method and tested them as photoanodes in DSSCs. The 4% Ag-doped TiO_2_ exhibited enhanced photovoltaic performance, with a *J*_SC_ of 8.336 mA cm^−2^, *V*_OC_ of 698 mV, FF of 0.422, and a PCE of 2.45%, significantly outperforming 2% Ag-doped TiO_2_ (PCE of 0.97%) and pristine TiO_2_ (PCE of 0.62%). Ag doping improved charge transport, reduced charge recombination, and enhanced the optical, structural, and electrochemical properties, leading to better device performance.^117^

Moreover, TiO_2_ doped with C and co-doped with Zn (Zn–C) and Sn (Sn–C) were prepared *via* sol–gel methods and tested as photoelectrodes in DSSCs by Nursam *et al.*^118^ The doping reduced TiO_2_ crystallite size and bandgap. The DSSC with undoped TiO_2_ achieved 3.83% efficiency, while C-doped TiO_2_ improved to 4.20%. However, Zn–C and Sn–C co-doped TiO_2_, resulting in lower efficiencies of 0.71% and 0.85%, respectively. Rajaramanan *et al.* present a method for fabricating nitrogen-doped TiO_2_ (N-doped TiO_2_) photoanodes for DSSC applications. N-doping was achieved by grinding NH_4_OH with P25–TiO_2_, followed by calcination at 500 °C. The optimized 20N–TiO_2_, with 20 μL NH_4_OH, showed a pore diameter of 15.99 nm and a reduced bandgap of 2.94 eV. The DSSC with 20N–TiO_2_ achieved a PCE of 6.16%, 20% higher than the control, attributed to increased dye uptake and reduced recombination.^119^

In conclusion, doping in TiO_2_ significantly enhances its optoelectronic properties, leading to improved charge transfer, reduced recombination, and extended light absorption, making it a highly effective strategy to boost the performance and efficiency of TiO_2_-based devices, particularly in DSSCs.

#### 6.4.2. Surface passivation

Surface treatment of TiO_2_ photoanodes is a key strategy for enhancing the efficiency and stability of DSSCs. TiO_2_, as the electron transport layer, faces challenges such as charge recombination, limited light absorption, and low electron mobility, which hinder overall performance. Surface treatments, including passivation, doping, and coating with advanced materials, help mitigate these issues by improving charge transport, reducing recombination, and extending light absorption into the visible spectrum. Techniques like TiCl_4_ passivation and MXene coatings create barriers that reduce electron–hole recombination, boosting *V*_OC_ and *J*_SC_.

Advanced nanomaterials, such as 2D-graphenes, MXenes, and plasmonic nanoparticles, enhance conductivity and light harvesting, leading to higher PCE. Additionally, these treatments improve DSSC stability by protecting TiO_2_ from degradation. Overall, surface-treated TiO_2_ photoanodes significantly enhance the photovoltaic performance and durability of DSSCs, making them crucial for advancing next-generation solar technologies. Recently, Tehare *et al.* synthesized rutile TiO_2_ photoanodes in copper containers that were spin-coated with passivation layers (MgO, CaCO_3_, ZrO_2_, SnO_2_). The MgO-passivated TiO_2_ electrode achieved the highest PCE of 6.05% and IPCE of 52%. Passivation increased electron lifetime from 0.48 ms to 0.65 ms and reduced charge transfer resistance from 36.9 Ω cm (pristine TiO_2_) to 20.16 Ω cm (MgO-passivated). The improvement is due to reduced charge recombination and enhanced electron mobility, as the passivation layers, especially MgO, act as barriers that prevent recombination and improve charge transport. Additionally, optimized energy level alignment and surface morphology enhance light harvesting and charge injection, leading to higher efficiency.^120^

In another report, Kumar *et al.* achieved a maximum photo-conversion efficiency of 9.84% using *o*-phenylenediamine (aromatic amines)-capped TiO_2_ composites as the scattering layer. The higher efficiency is attributed to the increased reflectivity of *o*-phenylenediamine-capped TiO_2_, which enhanced photon back-reflection to the photoanode, improving light absorption. Additionally, the carrier lifetime doubled from 4.29 ms (pristine TiO_2_) to 9.8 ms, reducing charge recombination and contributing to improved efficiency.^121^ Further, Babu *et al.* studied RF-sputtered WO_3_ for passivating TiO_2_ to reduce electron loss from surface defects. The WO_3_-coated TiO_2_ maintained dye adsorption and enhanced the efficiency of DSSCs by approximately 10% compared to pristine TiO_2_. This improvement is attributed to WO_3_ creating an energy barrier that promotes photo-electron injection while blocking reverse recombination. An optimal WO_3_ thickness was identified, as excessive coating reduced short-circuit current density by hindering electron injection. RF-sputtering proved effective for uniform, pinhole-free WO_3_ coatings, making it suitable for surface passivation in nanostructured photovoltaic devices.^122^

Kaliamurthy *et al.* introduced a DSSC design with 10% SrF_2_ incorporated with a 90% TiO_2_ photoanode. This configuration enhances charge separation and increases the dielectric constant (*ε*′ = 6.844 × 10^6^), improving charge transport and collection efficiency by optimizing dye loading and reducing charge recombination. The electrostatic shielding of SrF_2_ minimizes back electron transfer and passivates trap levels. As a result, the PCE improved to 7.58% compared to the reference DSSC, having a PCE of 6.99% under 1-sun illumination, reaching approximately 16.01% with an output power of 59.41 μW cm^−2^ under 1000 lx LED-5000 K illumination.^123^ Rodrigues *et al.* explored the use of polymeric co-adsorbents, poly 4-vinylbenzoic acid (PVBA) and poly(4-vinylpyridine) (P4VP), as alternatives to chenodeoxycholic acid (CDCA) in DSSCs. These polymers effectively suppress back electron transfer from the TiO_2_/N719 photoanode to the I_3_^−^/3I^−^ electrolyte, achieving 1-sun equivalent PCEs of 8.3% (PVBA) and 9% (P4VP), with artificial light PCEs of 17.5% and 22%, respectively. PVBA's carboxylic groups enhance adsorption at the photoanode–electrolyte interface, leading to over 1000 hours of stable PCE performance, comparable to CDCA. Time-correlated single photon counting photoluminescence spectroscopy confirmed improved charge injection and excited dye lifetimes, while intrinsic device degradation was assessed using the ISOS-L2 protocol.^124^ Singh *et al.* used 1-D graphene nanoribbons (GNRs) in TiO_2_ compact and mesoporous layers to enhance quantum dot-sensitized solar cells (QDSSCs) and photoelectrochemical (PEC) water-splitting performance. The optimal GNR content achieved a maximum PCE of 2.33% in QDSSCs and a photocurrent density of 1.92 mA cm^−2^ in PEC water splitting. Incorporating GNRs into the mesoporous layer further improved performance, resulting in a PCE of 3.06% for QDSSCs and 2.39 mA cm^−2^ for PEC water splitting. Overall, device optimization led to enhancements of 113% in PCE and 80% in photocurrent density, with co-sensitization yielding PCEs of 4.55% for QDSSCs and 2.67 mA cm^−2^ for PEC water splitting, demonstrating the effectiveness of GNRs in improving charge transport and reducing recombination.^125^

Hence, passivation in TiO_2_-based DSSCs significantly enhances device performance by mitigating charge recombination and improving charge transport, thereby increasing PCE. Future research should focus on exploring novel passivating agents, such as hybrid materials or self-assembled monolayers, to further optimise the interface between the TiO_2_ photoanode and the electrolyte. Additionally, integrating advanced characterization techniques can provide deeper insights into the mechanisms of passivation, enabling the development of more effective strategies for enhancing the stability and efficiency of DSSCs. Exploring environmentally friendly and cost-effective materials for passivation will also be crucial for the sustainable advancement of this technology.

#### 6.4.3. Composite (hybrid) electrodes

DSSCs offer a promising solution for sustainable energy conversion, but their PCE remains a critical challenge. Recent studies have explored innovative strategies to enhance photoanodes and counter electrodes (CEs) performance by incorporating advanced nanocomposite materials. Lemos *et al.*^126^ integrated Ti_3_C_2_T_*x*_ MXene with TiO_2_ to form hybrid photoanodes and demonstrated that adding 0.075 wt% Ti_3_C_2_T_*x*_ MXene flakes to TiO_2_ led to a 20% enhancement in PCE, attributed to improved electron transport and prolonged electron lifetimes. Density functional theory (DFT) calculations confirmed that the Ti_3_C_2_T_*x*_ MXene–TiO_2_ interface lowers the anatase conduction band potential, facilitating more efficient electron migration from the N719 dye to the output terminal. Additionally, better photocarrier separation was observed, further supporting the enhanced device performance. Later, Nagalingam *et al.*^127^ employed polyaniline (PANI)–modified Ti_3_C_2_T_*x*_ composites (PANI–Ti_3_C_2_T_*x*_) as CEs in DSSCs. The incorporation of PANI improved the electrical conductivity and surface wettability of Ti_3_C_2_T_*x*_, leading to increased electroactive sites and faster ion transport. Electrochemical analysis revealed that the PANI–Ti_3_C_2_T_*x*_-CE exhibited superior electrocatalytic activity and comparable charge transfer resistance to platinum, with a PCE of 6.9% under simulated sunlight. Notably, the PANI–Ti_3_C_2_T_*x*_-CE demonstrated exceptional stability and reproducibility, highlighting its potential as a cost-effective alternative to platinum-based electrodes.

Another study by Yan *et al.*^128^ focused on the co-introduction of Nb_2_O_5_ and Ti_3_C_2_ quantum dots (MQDs) into the photoanode. This combination significantly altered the energy level alignment, resulting in enhanced light absorption, improved carrier separation, and accelerated charge transfer. The DSSC fabricated with the Nb_2_O_5_–Ti_3_C_2_ MQD composite photoanode achieved a remarkable PCE of 7.24%, substantially higher than the standard device's efficiency of 4.60%. Nagalingam *et al.*^129^ again presented a poly(3,4-ethylene dioxythiophene)-decorated MXene (PEDOT@Ti_3_C_2_T_*x*_) composite as a cost-effective alternative to Pt-CEs in DSSCs. The 2D Ti_3_C_2_T_*x*_ MXene was synthesized *via* selective chemical etching, followed by the electro-polymerization of EDOT monomers. Under AM 1.5G illumination, the PEDOT@Ti_3_C_2_T_*x*_-CE achieved a PCE of 7.12%, compared to 8.7% for Pt-CE, outperforming PEDOT-only and Ti_3_C_2_T_*x*_-only CEs. The composite demonstrated enhanced charge transfer and mass transport capabilities. Stability tests over 15 days showed excellent performance retention and corrosion resistance, establishing PEDOT@Ti_3_C_2_T_*x*_ as a viable, scalable alternative to Pt for DSSC applications.

Lastly, a Ti_3_C_2_T_*x*_–polythiophene (Ti_3_C_2_T_*x*_–PTh) composite fabricated *via* interfacial polymerization demonstrated its effectiveness as a CE in DSSCs. Structural and electrochemical characterization revealed that the composite exhibited superior electrocatalytic activity and reduced charge transfer resistance compared to individual components. The DSSC using the Ti_3_C_2_T_*x*_–PTh CE achieved a PCE of 5.83%, comparable to that of traditional platinum-based CEs.^130^ These findings underscore the potential of Ti_3_C_2_T_*x*_-based composites to replace conventional platinum electrodes, offering a more sustainable and cost-effective solution for next-generation DSSCs. In summary, the comparative analysis of these studies highlights the versatility of Ti_3_C_2_T_*x*_ MXene in enhancing the performance of DSSCs through various modifications. The incorporation of conductive polymers like PANI and PTh, as well as the introduction of Nb_2_O_5_ and MQDs, significantly improves charge transport, carrier separation, and catalytic activity. These advancements position Ti_3_C_2_T_*x*_/TiO_2_-based composites as a promising alternative to traditional materials, paving the way for the development of high-efficiency, low-cost DSSCs with enhanced stability and scalability.

### 6.5 Charge transport and electronic analogy

1-D TiO_2_ nanostructures have distinct advantages over bulk TiO_2_ with a wide bandgap, including increased surface area, a large number of active sites, and the impact of quantum confinement phenomena. The main determinants of their electrical characteristics are the size, unique crystal facets, shape, and synchronization of these nanostructures, as well as their alignment. Quantum confinement factors cause the bandgap to widen when nanowire size approaches the nanoscale regime. Doping with foreign elements is the main tactic used to adjust the bandgap and realign the valence and conduction band configurations favourably with the sensitizer energy levels. With the use of this method, one can tune their electrical characteristics precisely, improving their functionality for use in DSSC. Vertically arranged nanowire-based photoanodes have been used to provide the most outstanding DSSC performances. In nanorods, a large decrease in the grain boundaries, which frequently serve as electron traps, results in a boost in the electron diffusion length and, as a result, better charge transfer. TNRs aspect ratio can be changed in order to change the bandgap. The conduction band edge shifts downward as the aspect ratio of nanorods rises. Similar to this, adding surface roughness and meso-porosity can increase the surface area of nanorods, further boosting their performance in DSSCs. These techniques are essential for enhancing the electrical characteristics and effectiveness of photoanodes based on nanorods for cutting-edge solar cell applications. Quantum confinement effects, an exciting phenomenon only seen in hydrothermal tubes, are shown in TNTs with a wall thickness below 5 nm. The abundance of oxygen vacancies and Ti^3+^ ions is another element enhancing charge transmission in these nanotubes. The potential of these nanotubes to transfer charges is significantly influenced by the annealing temperature. Numerous techniques, such as doping, surface adsorption, the creation of heterojunctions, and surface embellishment, can be used to design the bandgap in nanotubes. With the help of these techniques, the bandgap of the nanotubes may be precisely altered, affecting its electrical characteristics and, as a result, improving their efficiency in future applications, such as DSSC.^85,131–135^

**Fig. 18:**
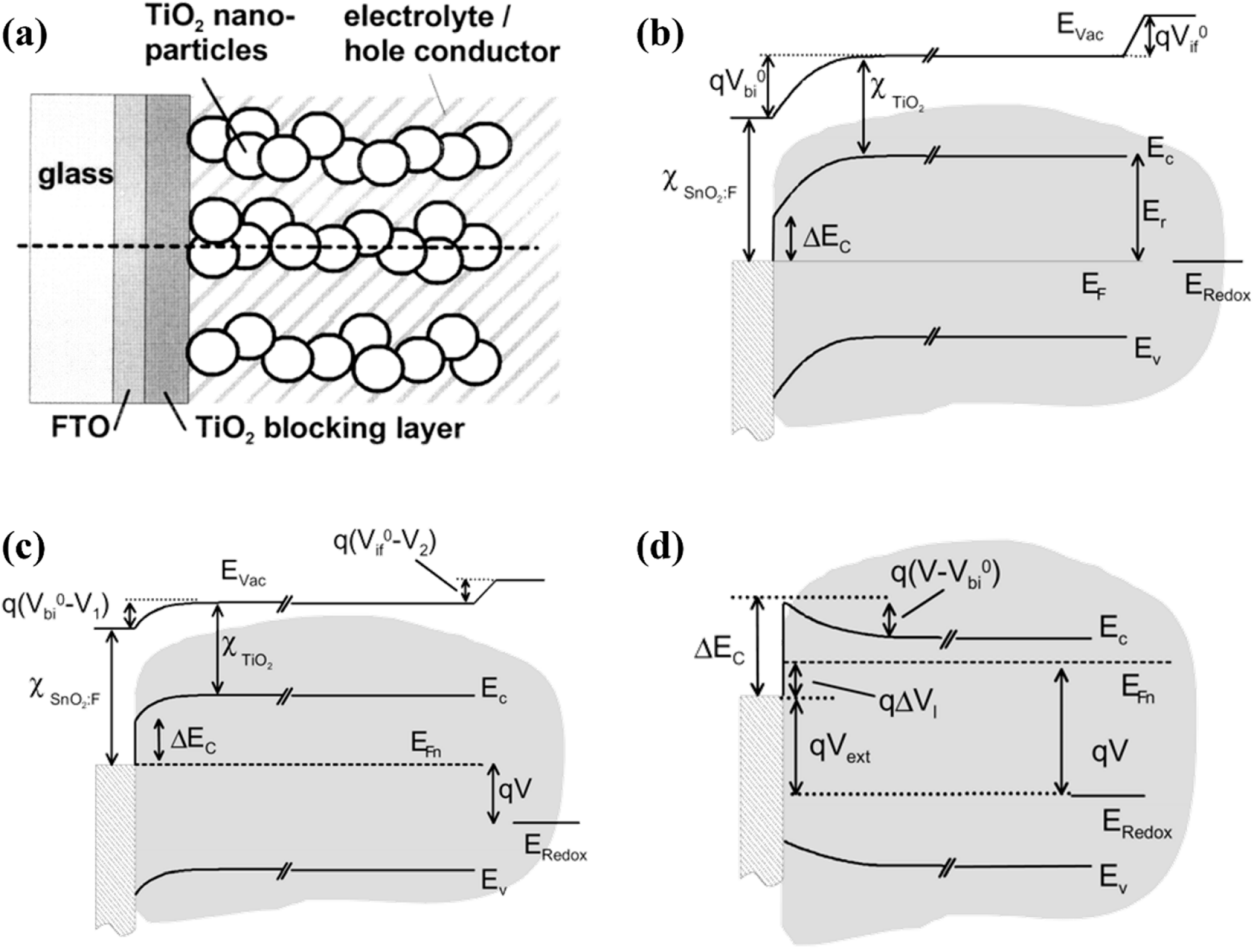
(a) Schematic side view of a DSSC comprising nanocrystalline 1-D TiO_2_ photoanodes and electrolyte, (b and c) schematic band model of DSSC under dark and light conditions, where the interfacial band bending arises due to the negative charges present in the electrolyte, and (d) detailed band diagram where light-induced *V* exceeds the *V*_bi_.

Despite ongoing debates on the exact mechanisms of photovoltaic action and the performance limitations of the original DSSC, there is consensus on the fundamental charge separation process upon illumination. This process involves the photoexcitation of the dye, the rapid injection of the excited electron into the TiO_2_ (on a timescale of <10^−12^ s), and the regeneration of the dye *via* electron transfer from the I^−^/I_3_^−^ redox couple, which occurs within 10 ns.^136–139^ The energy-band diagram of the electrolyte DSSC is well understood in thermal equilibrium despite the complex nanoscale mixture of TiO_2_ and electrolyte. The TiO_2_ network is idealized as columns (10–15 nm in diameter) on an FTO substrate or a TiO_2_ blocking layer, as shown in Fig. 18(a). Due to moderate doping, the charge within individual TiO_2_ particles is too small to cause significant band bending.^140^ The Helmholtz layer of the electrolyte screens any charge, preventing a substantial electric field along the TiO_2_ columns, as shown in the band diagram for the conduction and valence bands in Fig. 18(b).

At the TiO_2_-electrolyte interface, the equilibrium Fermi level (*E*_F_) is governed by the redox energy (*E*_redox_) of the electrolyte, which defines the potential at the interface.^141^ This is because the TiO_2_ is in contact with the electrolyte, and the Fermi level of TiO_2_ aligns with the redox potential of the electrolyte under equilibrium conditions. Additionally, an interface dipole forms at the TiO_2_-electrolyte interface due to several factors, including the polarity of the adsorbed dye, the Helmholtz layer of the electrolyte, and the differential adsorption of ions. Specifically, Li^+^ cations are adsorbed closer to the interface than I^−^ anions.^142,143^ This ion distribution results in a potential difference (*V*_if_) at the interface. This potential drop, *V*_if_, leads to the downward shift of the conduction band energy (*E*_CB_) of TiO_2_ relative to the *E*_redox_ of the electrolyte, creating a barrier that affects the charge transfer process. The energy alignment shown in Fig. 18(b), with the vacuum level (*E*_vac_), illustrates the influence of the dipole and potential drop at the interface, which plays a key role in determining the efficiency of charge separation and transport in DSSCs. An additional voltage drop, *V*_bi_, is required at the TiO_2_–SnO_2_/F interface to align the Fermi levels between the TiO_2_ and SnO_2_/F layers, ensuring proper energy level alignment, which is given below:

11 *qV*_bi_ = *E*_vac_ − *E*_redox_ − *χ*_SnO_2__ − *qV*_if_

where the electron affinity (*χ*_SnO_2__) of the SnO_2_/F plays a significant role in determining the position of the Fermi energy at the TiO_2_–SnO_2_/F interface In DSSCs, the built-in voltage (*V*_bi_) at the TiO_2_–SnO_2_/F interface is influenced by the charge distribution between the electrolyte and the highly doped SnO_2_/F layer.^144^ Negative charges from the electrolyte penetrate the TiO_2_ network, while positive charges from ionized SnO_2_/F atoms are confined to a thin layer near the TiO_2_ interface. This creates a potential drop (*V*_if_) across the TiO_2_-electrolyte interface, which is limited to the first one or two TiO_2_ nanograins due to the charge distribution geometry. The built-in voltage primarily affects the electron dynamics in these initial layers, with estimates of *V*_bi_ ranging from 0.3 V to 0.7 V, depending on material configuration and doping levels.^145,146^

The magnitude and significance of the *V*_bi_ in DSSCs remain subjects of debate. It is crucial to distinguish this from the separate question of whether DSSCs can be analyzed similarly to classical p–n-junction solar cells. Applying Shockley's p–n-junction theory can be useful for understanding DSSC behaviour, both in electrolyte-based and hole-conductor-based systems. However, experimental evidence does not suggest that the *V*_bi_ necessarily limits the *V*_OC_ of DSSCs, just as it does not strictly constrain p–n-junction solar cells.

Upon illumination, photoelectrons from the dye are injected into TiO_2_, causing a split in the Fermi level between TiO_2_ and the electrolyte, which drives the primary photovoltaic action in DSSCs. For external photovoltage measurement, the excess electrons in TiO_2_ must connect to the SnO_2_/F front electrode, while the redox potential of the redox couple must interface with the Pt back electrode. At open-circuit conditions, the photogenerated current density (*j*_ph_), balances the recombination current density (*j*_r_). Due to the extensive interface area between TiO_2_ and the electrolyte or hole conductor (HC), recombination mainly occurs at the TiO_2_ interface, involving oxidized redox species or, for solid-state DSSCs, holes from the HOMO. Cahen *et al.*^141^ identified the *V*_OC_, with the chemical potential difference, Δ*μ*, under these conditions, where *j*_r_ = *j*_ph_, highlighting the role of interfacial recombination in determining *V*_OC_. Based on Gerischer's semiconductor–electrolyte electron transfer theory, a general expression for the *V*_OC_ was formulated.^147–149^ This expression relates *V*_OC_ to the interfacial electron dynamics, encompassing the balance between the photogenerated electron density in the semiconductor and the redox energy level of the electrolyte, thereby providing insights into the voltage generation mechanism within DSSCs and given as,12
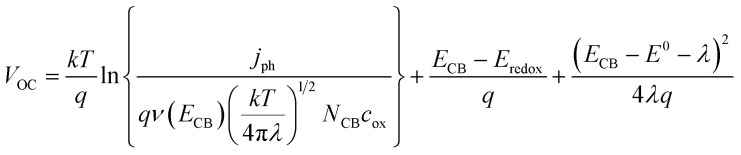
where *N*_CB_ is the effective density of states in TiO_2(CB)_, while *c*_ox_ denotes the concentration of oxidized species in the electrolyte. The term *ν*(*E*_CB_) describes the frequency factor for electron transfer from the semiconductor to the electrolyte at the conduction band edge energy. Additionally, *E*^0^ represents the standard redox potential as per Nernst's equation, and *λ* indicates the solvation reorganization energy. The derived expressions for *V*_OC_ assume that the excess charge carriers at the TiO_2_-electrolyte interface are governed by the externally applied voltage, whether this voltage drops across a space-charge region within TiO_2_ or across the SnO_2_/F–TiO_2_ interface. Furthermore, for solid-state DSSCs, *V*_OC_ is constrained by the recombination of photogenerated electrons in TiO_2_ with holes in the HC, as given:13
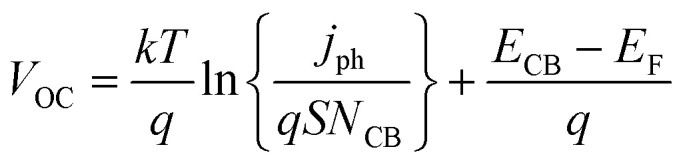
where *S* is interfacial recombination velocity.

When an external voltage drop occurs over the device, the effective band diagram must be adjusted from Fig. 18(b and c). This modifies the initial potential barriers *V*_bi_ and *V*_if_, with the externally measured or applied voltage *V* being the sum of *V*_1_ and *V*_2_. Consequently, this external voltage reduces one or both of these potential barriers according to,

14 *V*_bi_ + *V*_if_ = *V*_bi_^*V*=0^ − *V*_1_ + *V*_if_^*V*=0^ − *V*_2_ = *V*^0^_bi_ + *V*^0^_if_ − *V*

At zero applied voltage, the built-in voltage *V*_bi_^*V*=0^ and the interfacial potential *V*_if_^*V*=0^ remain at their initial values. When an external voltage is applied, *V*_bi_ decreases similarly to how the built-in potential of a bulk p–n-junction diode reduces under bias. However, the reduction of *V*_if_ is unique to DSSC and has no equivalent in conventional p–n-junction devices.

Zaban and co-workers noted that if the external voltage *V* only drops across the TiO_2_-electrolyte interface, reducing *V*_if_^*V*=0^ by *V* = *V*_2_ and *V*_1_ = 0, it would create an internal barrier, impeding electron injection into the TiO_2_ or dye regeneration from the electrolyte, or both.^142^ If *V* solely influences the *V*_bi_, the scenario where *V* exceeds *V*_bi_^*V*=0^ raises concerns. Photoelectrons in TiO_2_ would then need to overcome a potential barrier between the TiO_2_ and the SnO_2_/F window layer, which, while not necessarily affecting the open-circuit voltage *V*_OC_, could induce resistive losses, reducing the fill factor. Simulations by Ferber and Luther showed that even a low built-in voltage of 0.48 V could yield a *V*_OC_ around 0.78 V.^146^ This is consistent with theoretical constraints, as *V*_OC_ is limited by *V*_OC_ < *V*_bi_^*V*=0^ + Δ*E*_CB_, where Δ*E*_CB_ = *χ*_SnO_2__ − *χ*_TiO_2__. Nonetheless, the fill factor declines as *V*_bi_^*V*=0^ decreases from 0.66 to 0.48 V due to a loss-voltage Δ*V*_I_, which is necessary to push electrons over the electrostatic barrier (*V* − *V*_bi_^*V*=0^) into SnO_2_/F (Fig. 18(d)). Consequently, the usable external voltage *V*_ext_ = *V* − Δ*V*_I_, with Δ*V*_I_ being current-dependent and vanishing at *V*_ext_ = *V* = *V*_OC_.

The transport of electrons within the TiO_2_ network of DSSCs is governed by diffusion rather than drift, due to the absence of a significant electric field throughout most of the TiO_2_ structure. Under illumination or forward bias conditions (*V* < *V*_OC_), electron movement from the TiO_2_ nanoparticles to the front electrode primarily occurs through a diffusion-driven process. At moderate forward bias, the overall admittance of the DSSC is primarily influenced by the diffusion admittance of electrons in TiO_2_. According to the classical theory for single-sided p–n junctions described by Shockley, the time-dependent diffusion equation yields a complex expression for the diffusion admittance (*Y*_diff_) of minority carriers, particularly for voltages exceeding 3*kT*/*q*.^150^15
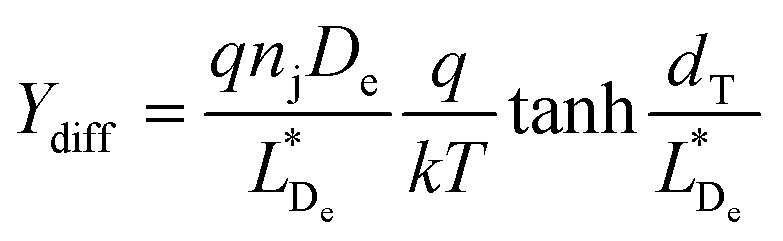
here, *D*_e_ represents the diffusion constant, while *n*_j_ denotes the excess electron density, defined as *n*_j_ = *n*_0_(exp(*qV*/(*kT*)) − 1), where *n*_0_ is the equilibrium electron density. The effective diffusion length, *L*_De_, is expressed as a complex quantity, *L*_De_(1 + *iωτ*)^−1/2^, which depends on the angular frequency *ω* = 2π*f*. Importantly, this formulation neglects electron recombination at the terminal of the TiO_2_ column with length *d*_T_.

The band model depicted in Fig. 18(b and c) applies to DSSCs with a liquid electrolyte. For solid-state DSSCs, two key distinctions must be addressed. Firstly, the Fermi energy level of the organic hole conductor differs from the redox potential of the I^−^/I_3_^−^ redox couple, with an energy difference of *E*_vac_ − *E*_redox_ = −4.95 eV.^151^ Secondly, in solid-state cells, the Fermi energy is influenced by the effective density of states in the valence band (*N*_V_), and the doping density as mentioned below:16
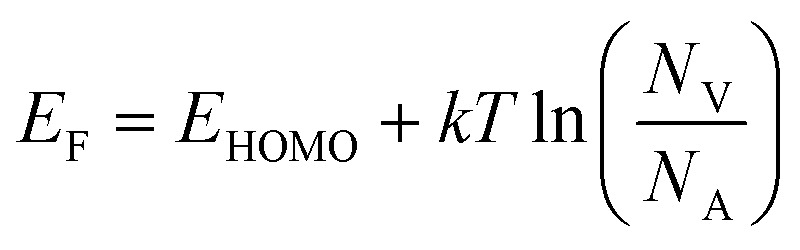


Using values of *N*_V_ ≈ 10^21^ cm^−3^ and *N*_A_ ≈ 10^17^ cm^−3^, the *E*_F_ of the HC layer is calculated to be 0.24 eV above the HOMO energy (*E*_HOMO_). The HOMO energy, measured *via* cyclic voltammetry, is approximately *E*_HOMO_ − *E*_vac_ = −4.8 eV in solution. Although the solid-state value may vary slightly, this estimation suggests a 0.2 eV difference between *E*_HOMO_ in the solid-state cell and the redox energy in the electrolyte-based cell. Another significant difference between solid-state and electrolyte DSSCs is the lower charge carrier density of the HC, compared to the high ion density in the redox electrolyte, resulting in a wider space charge region instead of a thin Helmholtz layer.^152^

## 7. Importance of 1-D TiO_2_ nanorods

Notably in the area of photovoltaics industry, 1-D TiO_2_ photoanodes are essential for energy conversion. They are important for a number of reasons.

### 7.1 Increased light absorption

Unlike to conventional TiO_2_ thin films, 1-D TiO_2_ nanostructures, such as nanowires, nanotubes, nanobelts or nanoneedles, offer an enormously huge surface area-to-volume ratio. This feature makes it feasible to use sunlight more effectively, improving the device's overall ability to convert photo energy into electrical energy.^153–155^

### 7.2 Superior charge transport

For photogenerated electrons and holes, 1-D TiO_2_ structures offer effective charge transport channels. Longer carrier lifetimes and larger photocurrents are produced as a consequence of the dearth of defects and grain boundaries in these structures. Higher energy conversion efficiencies are a result of this enhanced charge transport.^156–158^

### 7.3 Tunability of the bandgap

The 1-D structures' size and shape can be changed by doping *etc.* to alter the bandgap of TiO_2_. The bandgap can be tuned for better alignment with the solar spectrum and more effective solar energy harvesting.^159,160^

### 7.4 Stability and durability

1-D TiO_2_ structures display outstanding chemical stability and photo-corrosion resistance. This characteristic makes the photoanodes excellent for long-term energy conversion applications since it guarantees that they can endure repeated exposure to adverse weather conditions.^161–163^

### 7.5 Flexibility

1-D TiO_2_ structures are readily incorporated into a wide range of device topologies, including DSSCs, perovskite solar cells, and quantum dot solar cells. They are adaptable components in energy conversion systems because of their compatibility with various materials and fabrication processes.^22,164^

Moreover, other than application in photovoltaics, 1-D TiO_2_ nanostructures like nanotubes, nanowires, nanobelts, and nanorods, have been regarded as highly desirable candidates for a number of uses, including the photocatalytic degradation of pollutants, the photocatalytic conversion of CO_2_ into energy fuels, water splitting, supercapacitors, and lithium-ion batteries. Therefore, the remarkable properties of 1-D TiO_2_ nanostructures, ranging from enhanced light absorption and charge transport to tunability of bandgap and exceptional stability, position them as versatile and vital components not only in photovoltaics but also in a wide array of sustainable energy and environmental applications, showcasing their potential to drive advancements in renewable energy and catalysis technologies.

## 8. Current limitations and future perspective

DSSCs face critical challenges related to the electrolyte, Pt-counter electrode, and overall stability. The conventional I^−^/I_3_^−^ electrolyte suffers from volatility, leakage, and corrosiveness, leading to degradation of cell components and limiting long-term stability. Its narrow electrochemical window constrains the *V*_OC_, while slow redox species diffusion reduces the FF and overall efficiency. The high cost and scarcity of Pt, commonly used for its superior catalytic activity, significantly increase the production cost of DSSCs. Additionally, Pt degrades under exposure to the corrosive electrolyte, further impacting device longevity. These limitations have prompted research into cost-effective alternatives, such as carbon-based materials and transition metal compounds.^165,166^

Stability is another major concern, with TiO_2_ photoanodes susceptible to UV-induced degradation and dyes prone to photodegradation and thermal instability. The electrolyte also faces evaporation and leakage issues, reducing ionic conductivity over time. Effective encapsulation strategies are essential but challenging to implement without increasing costs. Current efforts to address these limitations include the development of solid-state electrolytes, alternative catalysts, and surface passivation techniques to enhance durability and efficiency, making DSSCs a more viable option for sustainable energy applications.^167–170^ Future evolutions in 1-D TiO_2_ nanostructures have enormous potential to influence the development of sustainable and energy-conversion technologies. Even more significant advancements in this area are probably possible with continued research and invention. These nanostructures might be crucial in solving crucial energy challenges.

### 8.1 Enhanced efficiency

We anticipate being able to precisely build 1-D TiO_2_ structures with respect to their attributes as our knowledge of nanoscale processes expands. This might lead to even better energy conversion efficiency, overcoming present constraints and boosting the affordability of renewable energy sources. Future approaches include integrating heterostructures and co-catalysts to improve charge separation, doping to extend light absorption, and surface engineering to minimize defect states. Scalable synthesis methods and AI-driven material optimization can further address challenges like recombination losses and cost barriers, positioning 1-D TiO_2_ as a key material for efficient and affordable renewable energy solutions.

### 8.2 Multifunctionality

Future research may unlock novel functionalities of 1-D TiO_2_ nano-structures, enabling them to serve as dual-purpose platforms for energy conversion and sensing. These advancements could facilitate their integration into smart energy grids for efficient energy management while also enabling real-time monitoring of environmental parameters such as pollutants, temperature, and humidity. By leveraging their photocatalytic and electronic properties, 1-D TiO_2_ structures could be tailored for applications in self-powered sensors, wearable devices, and Internet of Things (IoT) systems. Such multifunctionality would significantly broaden their impact across energy, environmental, and industrial sectors.

### 8.3 Integration and hybridization

Collaborative efforts to combine 1-D TiO_2_ nanostructures with advanced materials like perovskites, quantum dots, or MXenes could unlock synergistic effects, enhancing energy capture, transfer, and storage. By leveraging the unique properties of each material, such as the excellent charge transport of TiO_2_ and the superior light-harvesting capabilities of perovskites or quantum dots, these hybrid systems can achieve enhanced efficiency and flexibility. This integration may pave the way for innovative applications in next-generation solar cells, energy storage devices, and adaptable energy platforms for diverse environments.

### 8.4 Beyond photovoltaics

The versatility of 1-D TiO_2_ nanostructures could extend their application beyond photovoltaics into industries such as thermoelectrics. Their unique properties, including high surface area and tunable electronic characteristics, could be harnessed to efficiently convert waste heat into electricity, thereby improving energy efficiency. By integrating 1-D TiO_2_ nanostructures into thermoelectric devices, it may be possible to develop sustainable solutions for harnessing waste heat in industrial processes, transportation, and consumer electronics, contributing to energy conservation and reducing overall environmental impact.

### 8.5 Energy storage revolution

Integrating 1-D TiO_2_ nanostructures into energy storage systems, such as lithium-ion batteries and supercapacitors, can revolutionize energy storage technologies. These structures could significantly enhance the performance of these systems by improving charge storage capacity, increasing charge/discharge rates, and extending lifespan. The unique properties of 1-D TiO_2_, including high conductivity and stability, could enable faster charging times and more efficient energy retention. Moreover, their incorporation could improve the safety and reliability of energy storage devices, making them more suitable for a wide range of applications, from portable electronics to electric vehicles, while contributing to the overall advancement of sustainable energy solutions.

### 8.6 CO_2_ utilisation and catalysis

In the context of sustainable chemistry, 1-D TiO_2_ nanostructures hold significant potential as catalysts for the conversion of CO_2_ into valuable fuels or chemicals. Their photocatalytic properties could enable efficient solar-driven CO_2_ reduction processes, helping to mitigate greenhouse gas emissions while simultaneously producing useful by-products. By optimizing their surface properties and enhancing charge carrier dynamics, 1-D TiO_2_ could play a key role in advancing carbon-neutral technologies, contributing to the transition towards a society with minimal carbon emissions and a more sustainable approach to energy production and resource utilization.

### 8.7 Large-scale manufacturing

For 1-D TiO_2_ nanostructures to achieve widespread adoption, scaling up their production is crucial. The economic feasibility of these technologies will largely depend on the development of scalable, cost-effective manufacturing processes. Innovations in fabrication methods, such as continuous-flow synthesis, roll-to-roll processing, or green chemistry approaches, could enable large-scale production while maintaining the desired material properties. Additionally, reducing the cost of raw materials and energy-intensive steps will be key to making these advanced nanostructures accessible for commercial applications in energy, environmental, and industrial sectors, thereby accelerating their integration into sustainable technologies.

### 8.8 Global sustainability

Deploying 1-D TiO_2_ nanostructures in underdeveloped countries could provide sustainable and clean energy solutions in regions with limited or unreliable conventional energy infrastructure. These nanostructures could enable the creation of affordable, efficient energy systems, such as solar cells and energy storage devices, which are crucial for reducing dependence on fossil fuels and improving energy access. By facilitating the transition to renewable energy, 1-D TiO_2_ nanostructures could contribute to global sustainability efforts, ensuring equitable access to energy for all while also supporting the fight against climate change and fostering economic development in underserved communities.

Fundamentally, 1-D TiO_2_ nanostructures have a bright future in energy conversion and sustainable technology. These structures have the potential to alter our energy environment and usher in a more sustainable and resilient future as interdisciplinary collaborations bloom and as our understanding of nanomaterials advances.

## 9. Conclusion

This review provides an in-depth exploration of 1-D TiO_2_-nanostructured photoanode materials used in DSSCs, examining the intricate designs and operational principles of various 1-D TiO_2_ nanostructures. The shift towards renewable energy becomes increasingly critical as the world continues to rely heavily on non-renewable energy sources, leading to significant environmental damage and resource depletion. Solar energy, in particular, stands out as an abundant and sustainable source, offering the potential for both electricity and hydrogen fuel production. DSSCs, as efficient energy converters, are emerging as a promising solution to address the global energy crisis. Recent advancements in 1-D TiO_2_ nanostructures, such as nanoparticles, nanowires, nanorods, and nanotubes, have made them ideal candidates for high-performance DSSC photoanodes, owing to their superior light-scattering properties and rapid electron transport capabilities. Overcoming surface area limitations holds promise for even greater efficiency, positioning 1-D TiO_2_ as a more attractive option than other semiconductors due to its well-matched band structure with the sensitizer and efficient charge transport. Furthermore, 1-D TiO_2_'s non-toxic nature and robust functionality enhance its appeal in sustainable energy applications.

Future research may focus on extending the absorption spectrum of 1-D TiO_2_ into the infrared (IR) region to further enhance charge collection and broaden its applicability under diverse lighting conditions. The integration of 1-D TiO_2_ with other advanced materials, such as perovskites, quantum dots, and MXenes, could enable synergies that maximize energy capture, transfer, and storage. Additionally, the exploration of 1-D TiO_2_ nanostructures in energy storage systems, CO_2_ conversion technologies, and underdeveloped regions could further contribute to a sustainable global energy transition. By overcoming current challenges in scalability, surface engineering, and hybridization, 1-D TiO_2_-based DSSCs can play a pivotal role in the future of clean energy solutions, advancing both energy efficiency and environmental sustainability.

## Data availability

All data used will be made available by the corresponding author on reasonable request.

## Author contributions

Kumar Vaisno Srivastava: conceptualization, visualization, validation, investigation, methodology, formal analysis, writing – original draft, writing – review & editing, and resources. Pooja Srivastava: writing – review & editing. Akancha Srivastava: formal analysis. Raj Kumar Maurya: supervision. Yatendra Pal Singh: supervision. Abhishek Srivastava: conceptualization, visualization, validation, investigation, methodology, formal analysis, writing – original draft, writing – review & editing, resources, supervision.

## Conflicts of interest

There is no conflict to declare.

## References

[cit1] Pietro Colelli F., Emmerling J., Marangoni G., Mistry M. N., De Cian E. (2022). Nat. Commun..

[cit2] L.-E. British Oil and BP Gas Company , Statistical Review of World Energy, 2017

[cit3] Green M., Dunlop E., Hohl-Ebinger J., Yoshita M., Kopidakis N., Hao X. (2021). Prog. Photovoltaics Res. Appl..

[cit4] Grätzel M. (2003). J. Photochem. Photobiol. C Photochem. Rev..

[cit5] Freitag M., Teuscher J., Saygili Y., Zhang X., Giordano F., Liska P., Hua J., Zakeeruddin S. M., Moser J. E., Grätzel M., Hagfeldt A. (2017). Nat. Photonics.

[cit6] O'Regan B., Grätzel M. (1991). et al.. Nature.

[cit7] Kakiage K., Aoyama Y., Yano T., Oya K., Fujisawa J. I., Hanaya M. (2015). Chem. Commun..

[cit8] Tamilselvan S. N., Shanmugan S. (2024). Clean Energy.

[cit9] Tang Z., Wu J., Zheng M., Huo J., Lan Z. (2013). Nano Energy.

[cit10] Garnett E., Mai L., Yang P. (2019). Chem. Rev..

[cit11] Nehra M., Dilbaghi N., Marrazza G., Kaushik A., Abolhassani R., Mishra Y. K., Kim K. H., Kumar S. (2020). Nano Energy.

[cit12] Yun H. J., Lee H., Joo J. B., Kim W., Yi J. (2009). J. Phys. Chem. C.

[cit13] Razzaq A., Allen T. G., Liu W., Liu Z., De Wolf S. (2022). Joule.

[cit14] Li X., Yang Y., Huang S., Jiang K., Li Z., Zhao W., Yu J., Gao Q., Han A., Shi J., Du J., Meng F., Zhang L., Liu Z., Liu W. (2022). Sol. Energy Mater. Sol. Cells.

[cit15] Becker C., Amkreutz D., Sontheimer T., Preidel V., Lockau D., Haschke J., Jogschies L., Klimm C., Merkel J. J., Plocica P., Steffens S., Rech B. (2013). Sol. Energy Mater. Sol. Cells.

[cit16] Lee T. D., Ebong A. U. (2017). Renew. Sustain. Energy Rev..

[cit17] Ilic S., Paunovic V. (2019). Facta Univ. – Ser. Electron. Energ..

[cit18] Tributsch H. (2004). Coord. Chem. Rev..

[cit19] Zatirostami A. (2020). J. Alloys Compd..

[cit20] Luque A. S. H. , Handbook of Photovoltaic Science and Engineering, 2011

[cit21] Sugathan V., John E., Sudhakar K. (2015). Renew. Sustain. Energy Rev..

[cit22] Sharma K., Sharma V., Sharma S. S. (2018). Nanoscale Res. Lett..

[cit23] Katoh R., Furube A. (2014). J. Photochem. Photobiol. C Photochem. Rev..

[cit24] Anta J. A., Mora-Seró I., Dittrich T., Bisquert J. (2007). J. Phys. Chem. C.

[cit25] Deb Nath N. C., Lee J. J. (2019). J. Ind. Eng..

[cit26] Frank A. J., Kopidakis N., Van De Lagemaat J. (2004). Coord. Chem. Rev..

[cit27] Uddin M. G. , Development of Simplified In Situ Processing Routes for Rear-Side Patterning of Silicon Heterojunction Interdigitated Back Contact (SHJ-IBC) Solar Cells, M.Sc. thesis, University of Eastern Finland, 2018, p. 90

[cit28] Zhu K., Kopidakis N., Neale N. R., Van De Lagemaat J., Frank A. J. (2006). J. Phys. Chem. B.

[cit29] De Angelis F., Fantacci S., Selloni A. (2008). Nanotechnology.

[cit30] Zhu K., Neale N. R., Miedaner A., Frank A. J. (2007). Nano Lett..

[cit31] Kumari J. M. K. W., Sanjeevadharshini N., Dissanayake M. A. K. L., Senadeera G. K. R., Thotawatthage C. A. (2016). Ceylon J. Sci..

[cit32] Wang P., Zakeeruddin S. M., Humphry-Baker R., Moser J. E., Grätzel M. (2003). Adv. Mater..

[cit33] Cameron P. J., Peter L. M. (2003). J. Phys. Chem. B.

[cit34] Van De Lagemaat J., Kopidakis N., Neale N. R., Frank A. J. (2005). Phys. Rev. B – Condens. Matter Mater. Phys..

[cit35] Pace S., Resmini A., Tredici I. G., Soffientini A., Li X., Dunn S., Briscoe J., Anselmi-Tamburini U. (2018). RSC Adv..

[cit36] Ju M. G., Chen M., Zhou Y., Garces H. F., Dai J., Ma L., Padture N. P., Zeng X. C. (2018). ACS Energy Lett..

[cit37] Yu X., Zeng W. (2016). J. Mater. Sci. Mater. Electron..

[cit38] Prabavathy N., Shalini S., Balasundaraprabhu R., Velauthapillai D., Prasanna S., Walke P., Muthukumarasamy N. (2017). J. Mater. Sci. Mater. Electron..

[cit39] Corby S., Rao R. R., Steier L., Durrant J. R. (2021). Nat. Rev. Mater..

[cit40] Tahir M. B. , Rafique M., Rafique M. S., Fatima N. and Israr Z., Metal Oxide- and Metal Sulfide-Based Nanomaterials as Photocatalysts, Elsevier Inc., 2020

[cit41] Lee C. P., Li C. T., Ho K. C. (2017). Mater. Today.

[cit42] Bhojanaa A. P. K. B., Soundarya Mary A., Shalini Devi K. S., Pavithra N. (2022). Sol. RRL.

[cit43] Baig N., Kammakakam I., Falath W., Kammakakam I. (2021). Mater. Adv..

[cit44] Low F. W., Lai C. W. (2018). Renew. Sustain. Energy Rev..

[cit45] Al A., Mashfiqua R., Datta D., Ghosh S., Datta O., Molla A., Begum M. E. (2024). Heliyon.

[cit46] Yang Z., Ma Z., Ren J., Xiong Y., Ren F. (2024). Opt. Mater..

[cit47] Cui H., Liu H., Shi J., Wang C. (2013). Int. J. Photoenergy.

[cit48] Zhao W., Guo P., Wu J., Lin D., Jia N., Fang Z., Liu C., Ye Q., Zou J., Zhou Y., Wang H. (2024). Nano–Micro Lett..

[cit49] Zhen C., Wu T., Chen R., Wang L., Liu G., Cheng H. M. (2019). ACS Sustain. Chem. Eng..

[cit50] Kim Y. S., Jin H. J., Jung H. R., Kim J., Nguyen B. P., Kim J., Jo W. (2021). Sci. Rep..

[cit51] Bourikas K., Kordulis C., Lycourghiotis A. (2014). Chem. Rev..

[cit52] Scanlon D. O., Dunnill C. W., Buckeridge J., Shevlin S. A., Logsdail A. J., Woodley S. M., Catlow C. R. A., Powell M. J., Palgrave R. G., Parkin I. P., Watson G. W., Keal T. W., Sherwood P., Walsh A., Sokol A. A. (2013). Nat. Mater..

[cit53] Kim S. A., Hussain S. K., Abbas M. A., Bang J. H. (2022). J. Solid State Chem..

[cit54] Siddiqui H. , Ion Beam Techniques and Applications, Intech, 2019, 10.5772/intechopen.83566

[cit55] Zatirostami A. (2021). Opt. Mater..

[cit56] Mukametkali T. M., Ilyassov B. R., Aimukhanov A. K., Serikov T. M., Baltabekov A. S., Aldasheva L. S., Zeinidenov A. K. (2023). Phys. B Condens. Matter.

[cit57] Zhang W., Liu Y., Zhou D., Wang H., Liang W., Yang F. (2016). Beilstein J. Nanotechnol..

[cit58] Jin S., Shin E., Hong J. (2017). Nanomaterials.

[cit59] Yang H. G., Liu G., Qiao S. Z., Sun C. H., Jin Y. G., Smith S. C., Zou J., Cheng H. M., Lu G. Q. (2009). J. Am. Chem. Soc..

[cit60] Shinde P. V., Gagare S., Rout C. S., Late D. J. (2020). RSC Adv..

[cit61] Liu J., Zhang Q., Niu L., Hu B., Zhou X. (2016). J. Mater. Sci. Mater. Electron..

[cit62] Hurum D. C., Agrios A. G., Gray K. A., Rajh T., Thurnauer M. C. (2003). J. Phys. Chem. B.

[cit63] Landmann M., Rauls E., Schmidt W. G. (2012). J. Phys. Condens. Matter.

[cit64] Zavabeti A. , Jannat A., Zhong L., Haidry A. A., Yao Z. and Ou J. Z., Two-Dimensional Materials in Large-Areas: Synthesis, Properties and Applications, Springer Singapore, 2020, vol. 12 10.1007/s40820-020-0402-x PMC7770797 34138280

[cit65] Shanmugam V., Mensah R. A., Babu K., Gawusu S., Chanda A., Tu Y., Neisiany R. E., Försth M., Sas G., Das O. (2022). Part. Part. Syst. Charact..

[cit66] Da Silva A. F., Dantas N. S., Da Silva E. F., Pepe I., Torres M. O., Persson C., Lindgren T., De Almeida J. S., Ahuja R. (2005). AIP Conf. Proc..

[cit67] Guo W., Shen Y., Boschloo G., Hagfeldt A., Ma T. (2011). Electrochim. Acta.

[cit68] Iijima S. (1991). Nature.

[cit69] Law M., Greene L. E., Johnson J. C., Saykally R., Yang P. (2005). Nat. Mater..

[cit70] Joshy D., Narendranath S. B., Ismail Y. A., Periyat P. (2022). Nanoscale Adv..

[cit71] Feng X., Shankar K., Varghese O. K., Paulose M., Latempa T. J., Grimes C. A. (2008). Nano Lett..

[cit72] Liu W., Lu H., Zhang M., Guo M. (2015). Appl. Surf. Sci..

[cit73] Bakhshayesh A. M., Mohammadi M. R., Dadar H., Fray D. J. (2013). Electrochim. Acta.

[cit74] Zha C., Shen L., Zhang X., Wang Y., Korgel B. A., Gupta A., Bao N. (2014). ACS Appl. Mater. Interfaces.

[cit75] Wu W. Q., Xu Y. F., Su C. Y., Bin Kuang D. (2014). Energy Environ. Sci..

[cit76] Yen Y. C., Chen P. H., Chen J. Z., Chen J. A., Lin K. J. (2015). ACS Appl. Mater. Interfaces.

[cit77] Wu W. Q., Xu Y. F., Rao H. S., Su C. Y., Bin Kuang D. (2014). J. Am. Chem. Soc..

[cit78] Roh D. K., Chi W. S., Ahn S. H., Jeon H., Kim J. H. (2013). ChemSusChem.

[cit79] Li H., Yu Q., Huang Y., Yu C., Li R., Wang J., Guo F., Jiao S., Gao S., Zhang Y., Zhang X., Wang P., Zhao L. (2016). ACS Appl. Mater. Interfaces.

[cit80] Ni S., Wang D., Guo F., Jiao S., Zhang Y., Wang J., Wang B., Yuan L., Zhang L., Zhao L. (2019). J. Cryst. Growth.

[cit81] Jiu J., Isoda S., Wang F., Adachi M. (2006). J. Phys. Chem. B.

[cit82] De Marco L., Manca M., Giannuzzi R., Malara F., Melcarne G., Ciccarella G., Zama I., Cingolani R., Gigli G. (2010). J. Phys. Chem. C.

[cit83] Zhang W., Xie Y., Xiong D., Zeng X., Li Z., Wang M., Cheng Y. B., Chen W., Yan K., Yang S. (2014). ACS Appl. Mater. Interfaces.

[cit84] Liu J., Luo J., Yang W., Wang Y., Zhu L., Xu Y., Tang Y., Hu Y., Wang C., Chen Y., Shi W. (2015). J. Mater. Sci. Technol..

[cit85] Lee B. H., Song M. Y., Jang S. Y., Jo S. M., Kwak S. Y., Kim D. Y. (2009). J. Phys. Chem. C.

[cit86] Yang L., Leung W. W. F. (2013). Adv. Mater..

[cit87] Chen X., Du Q., Yang W., Liu W., Miao Z., Yang P. (2018). J. Solid State Electrochem..

[cit88] Subramaniam M. R., Kumaresan D., Jothi S., McGettrick J. D., Watson T. M. (2018). Appl. Surf. Sci..

[cit89] Tang Y., Wang C., Hu Y., Huang L., Fu J., Yang W. (2016). Superlattices Microstruct..

[cit90] Jiu J., Wang F., Isoda S., Adachi M. (2005). Chem. Lett..

[cit91] Chatterjee S., Webre W. A., Patra S., Rout B., Glass G. A., D'Souza F., Chatterjee S. (2020). J. Alloys Compd..

[cit92] Chen H. Y., Zhang T. L., Fan J., Bin Kuang D., Su C. Y. (2013). ACS Appl. Mater. Interfaces.

[cit93] Rui Y., Li Y., Zhang Q., Wang H. (2013). Nanoscale.

[cit94] Kathirvel S., Su C., Shiao Y. J., Lin Y. F., Chen B. R., Li W. R. (2016). Sol. Energy.

[cit95] Sriharan N., Senthil T. S., Kang M., Ganesan N. M. (2019). Appl. Phys. A Mater. Sci. Process..

[cit96] Liu N., Chen X., Zhang J., Schwank J. W. (2014). Catal. Today.

[cit97] Cheng H., Feng Y., Fu Y., Zheng Y., Shao Y., Bai Y. (2022). J. Mater. Chem. C.

[cit98] Wang J., Lin Z. (2010). Chem. Mater..

[cit99] Lei B. X., Liao J. Y., Zhang R., Wang J., Su C. Y., Bin Kuang D. (2010). J. Phys. Chem. C.

[cit100] Park H., Kim W. R., Jeong H. T., Lee J. J., Kim H. G., Choi W. Y. (2011). Sol. Energy Mater. Sol. Cells.

[cit101] Roy P., Kim D., Paramasivam I., Schmuki P. (2009). Electrochem. commun..

[cit102] He Z., Que W., Sun P., Ren J. (2013). ACS Appl. Mater. Interfaces.

[cit103] Fu N., Jiang X., Chen D., Duan Y., Zhang G., Chang M., Fang Y., Lin Y. (2019). J. Power Sources.

[cit104] Bozkurt Çırak B., Eden Ç., Erdoğan Y., Demir Z., Özdokur K. V., Caglar B., Morkoç Karadeniz S., Kılınç T., Ercan Ekinci A., Çırak Ç. (2020). Optik.

[cit105] Wu W. Q., Xu Y. F., Rao H. S., Su C. Y., Bin Kuang D. (2014). J. Phys. Chem. C.

[cit106] Abdullah M., Kamarudin S. K. (2017). Renew. Sustain. Energy Rev..

[cit107] Adachi M., Murata Y., Okada I., Yoshikawa S. (2003). J. Electrochem. Soc..

[cit108] Zheng Q., Kang H., Yun J., Lee J., Park J. H., Baik S. (2011). ACS Nano.

[cit109] Hu J. H., Tong S. Q., Yang Y. P., Cheng J. J., Zhao L., Duan J. X. (2016). Acta Metall. Sin..

[cit110] Khan J., Gu J., He S., Li X., Ahmed G., Liu Z., Akhtar M. N., Mai W., Wu M. (2017). Nanoscale.

[cit111] Zhu W., Liu Y., Yi A., Zhu M., Li W., Fu N. (2019). Electrochim. Acta.

[cit112] Asahi R., Taga Y., Mannstadt W. (2000). Phys. Rev. B – Condens. Matter Mater. Phys..

[cit113] Umebayashi T., Yamaki T., Itoh H., Asai K. (2002). J. Phys. Chem. Solids.

[cit114] Khlyustova A., Sirotkin N., Kusova T., Kraev A., Titov V., Agafonov A. (2020). Mater. Adv..

[cit115] Ju H.-W., Kwon J.-W., Lee D.-N., Park Y.-J., Lee S.-E., Kim T. (2024). Adv. Power Technol..

[cit116] Ayaz M., Alatawi A. S., Hijji M., Namazi M. A., Ershath M. I. M. (2024). J. Phys. Chem. Solids.

[cit117] Sharif A. M., Ashrafuzzaman M., Kalam A., Al-Sehemi A. G., Yadav P., Tripathi B., Dubey M., Du G. (2023). Materials.

[cit118] Novianti R. M., Nursam N. M., Shobih S., Hidayat J., Soepriyanto S. (2023). Metalurgi.

[cit119] Rajaramanan T., Heidari Gourji F., Velauthapillai D., Ravirajan P., Senthilnanthanan M. (2023). Int. J. Energy Res..

[cit120] Tehare K. K., Navale S. T., Stadler F. J., He Z., Yang H., Xiong X., Liu X., Mane R. S. (2018). Mater. Res. Bull..

[cit121] Naveen Kumar T. R., Yuvaraj S., Kavitha P., Sudhakar V., Krishnamoorthy K., Neppolian B. (2020). Sol. Energy.

[cit122] Babu A., Vasanth A., Nair S., Shanmugam M. (2021). J. Semicond..

[cit123] Kaliamurthy A. K., Asiam F. K., Yadagiri B., Chen C., Kang H. C., Sandhu S., Qamar M. Z., Yoo K., Lee J. J. (2024). ACS Appl. Energy Mater..

[cit124] Rodrigues D. F. S. L., Martins J., Sauvage F., Abreu C. M. R., Coelho J. F. J., Serra A. C., Ivanou D., Mendes A. (2024). Surfaces and Interfaces.

[cit125] Singh I., Bhullar V., Mahajan A. (2023). Energy and Fuels.

[cit126] Lemos H. G., Ronchi R. M., Portugal G. R., Rossato J. H. H., Selopal G. S., Barba D., Venancio E. C., Rosei F., Arantes J. T., Santos S. F. (2022). ACS Appl. Energy Mater..

[cit127] Nagalingam S. P., Pandiaraj S., Alodhayb A. N., Grace A. N. (2024). Nanoscale.

[cit128] Yan H., Chen M., Liu W., Wang P., Liu M., Liu Y., Ye L., Gu M. (2023). Opt. Mater..

[cit129] Priya Nagalingam S., Grace A. N. (2022). Mater. Today Chem..

[cit130] Nagalingam S. P., Pandiaraj S., Alzahrani K. E., Alodhayb A. N., Grace A. N. (2024). RSC Adv..

[cit131] Wang H., Liu M., Zhang M., Wang P., Miura H., Cheng Y., Bell J. (2011). Phys. Chem. Chem. Phys..

[cit132] Yang M., Kim D., Jha H., Lee K., Paul J., Schmuki P. (2011). Chem. Commun..

[cit133] So S., Lee K., Schmuki P. (2012). Phys. Status Solidi - Rapid Res. Lett..

[cit134] De Marco L., Manca M., Giannuzzi R., Belviso M. R., Cozzoli P. D., Gigli G. (2013). Energy Environ. Sci..

[cit135] Lee K., Mazare A., Schmuki P. (2014). Chem. Rev..

[cit136] Tachibana Y., Moser J. E., Grätzel M., Klug D. R., Durrant J. R. (1996). J. Phys. Chem..

[cit137] Cherepy N. J., Smestad G. P., Grätzel M., Zhang J. Z. (1997). J. Phys. Chem. B.

[cit138] Hilgendorff M., Sundström V. (1998). J. Phys. Chem. B.

[cit139] Hagfeldt A., Grätzel M. (2000). Acc. Chem. Res..

[cit140] O'Regan B., Moser J., Anderson M., Grätzel M. (1990). J. Phys. Chem..

[cit141] Cahen D., Hodes G., Grätzel M., Guillemoles J. F., Riess I. (2000). J. Phys. Chem. B.

[cit142] Zaban A., Ferrere S., Gregg B. A. (1998). J. Phys. Chem. B.

[cit143] Zaban A., Meier A., Gregg B. A. (1997). J. Phys. Chem. B.

[cit144] Schwarzburg K., Willig F. (1999). J. Phys. Chem. B.

[cit145] Bisquert J., Garcia-Belmonte G., Fabregat-Santiago F. (1999). J. Solid State Electrochem..

[cit146] Ferber J., Luther J. (2002). J. Phys. Chem. B.

[cit147] Gerischer H. , Physical Chemistry: An Advanced Treatise, Academic Press-New York-London, 1970, pp. 487–489

[cit148] Rosenbluth M. L., Lewis N. S. (1989). J. Phys. Chem..

[cit149] Pleskov J. , Gurevich Y. and Bartlett P., Semiconductor Photoelectrochemistry, New York Consulting Bureau, 1986, pp. 135–138

[cit150] Shockley W. (1949). Bell Syst. Tech. J..

[cit151] Hagfeldtt A., Gratzel M. (1995). Chem. Rev..

[cit152] Kron G., Egerter T., Werner J. H., Rau U. (2003). J. Phys. Chem. B.

[cit153] Soussi A., Ait Hssi A., Boujnah M., Boulkadat L., Abouabassi K., Asbayou A., Elfanaoui A., Markazi R., Ihlal A., Bouabid K. (2021). J. Electron. Mater..

[cit154] Evtushenko Y. M., Romashkin S. V., Trofimov N. S., Chekhlova T. K. (2015). Phys. Procedia.

[cit155] Khan M. I., Bhatti K. A., Qindeel R., Althobaiti H. S., Alonizan N. (2017). Results Phys..

[cit156] Qian R., Zong H., Schneider J., Zhou G., Zhao T., Li Y., Yang J., Bahnemann D. W., Pan J. H. (2019). Catal. Today.

[cit157] Yang F., Xi J., Gan L. Y., Wang Y., Lu S., Ma W., Cai F., Zhang Y., Cheng C., Zhao Y. (2016). J. Colloid Interface Sci..

[cit158] Al Mayyahi A., Everhart B. M., Shrestha T. B., Back T. C., Amama P. B. (2021). RSC Adv..

[cit159] Wadhwa P., Kumar S., Kumar T. J. D., Shukla A., Kumar R. (2018). Condens. Matter.

[cit160] Singh Surah S., Vishwakarma M., Kumar R., Nain R., Sirohi S., Kumar G. (2019). Results Phys..

[cit161] Minhas B., Dino S., Qian H., Zuo Y. (2020). Surf. Coatings Technol..

[cit162] Mowbray D. J., Martinez J. I., Lastra G. J. M., Thygesen K. S., Jacobsen K. W. (2009). J. Phys. Chem. C.

[cit163] Das P. K., Mallik A. K., Molla A. H., Santra A. K., Ganguly R., Saha A., Kumar S., Aswal V. K. (2022). J. Therm. Anal. Calorim..

[cit164] Poh S. C., Ahmad H., Ting C. H., Tung H. T., Jun H. K. (2021). J. Mater. Sci. Mater. Electron..

[cit165] Gong J., Sumathy K., Qiao Q., Zhou Z. (2017). Renew. Sustain. Energy Rev..

[cit166] Mozaffari S., Nateghi M. R., Zarandi M. B. (2017). Renew. Sustain. Energy Rev..

[cit167] Chen T., Xie J., Gao P. (2022). Adv. Energy Sustain. Res..

[cit168] Groeneveld I., Kanelli M., Ariese F., van Bommel M. R. (2023). Dye. Pigment..

[cit169] Reza K. M., Kurny A., Gulshan F. (2017). Appl. Water Sci..

[cit170] Sekaran P. D., Marimuthu R. (2024). Brazilian J. Phys..

